# Viewers Actively Extract and Maintain Spontaneous Theory of Mind Representations in Dramatic Irony Scenes

**DOI:** 10.1111/cogs.70273

**Published:** 2026-10-30

**Authors:** Cynthia Cabañas, Sam Boland, Atsushi Senju, Tim J. Smith

**Affiliations:** ^1^ Department of Communication Science Vrije Universiteit Amsterdam; ^2^ Department of Psychological Sciences Birkbeck, University of London; ^3^ Research Centre for Child Mental Development Hamamatsu University School of Medicine; ^4^ Creative Computing Institute University of the Arts London

**Keywords:** Dramatic irony, Spontaneous Theory of Mind, False‐belief attribution, Event comprehension, Visual attention, Eye‐tracking

## Abstract

Understanding others’ mental states is a cornerstone of human cognition, yet how spontaneous belief attribution operates in complex, naturalistic perception remains underexplored. Dramatic irony (DI)—a narrative device through which viewers are aware of information unknown to characters—provides a controlled but naturalistic context to examine these processes. Using a curated DI film corpus, we combined eye‐tracking with (a) self‐paced and (b) video viewing to investigate how Spontaneous Theory of Mind shapes real‐time processing. Across two experiments, participants viewed edited Harold Lloyd silent film excerpts that either provided privileged information (Installation condition) or aligned viewer and character knowledge (Control condition), reflecting false‐ and true‐belief scenarios. Clips were either presented as a self‐paced static series of frames (Experiment 1) or dynamic film clips (Experiment 2). We analyzed narrative comprehension, reaction times, and eye‐tracking measures indexing cognitive effort. Results showed that, relative to the Control condition, the Installation condition yielded longer viewing times at moments of representational conflict, indicating increased cognitive demands associated with maintaining and applying false‐belief representations. In Experiment 1, these effects emerged at discrete moments and were accompanied by longer fixation durations in the Installation condition. In Experiment 2, longer fixation durations in the Installation condition were again observed, alongside reduced fixation rates and saccade amplitudes, consistent with more sustained local processing. These findings indicate that viewers spontaneously construct and maintain false‐belief representations during DI, which modulate real‐time processing during scene comprehension, providing a tractable framework for investigating social reasoning in dynamic visual contexts.

## 1 Introduction

When watching films, viewers actively interpret events by inferring characters’ intentions and motives. This involves spontaneously considering what characters perceive and know, forming the basis for predictions about their behavior. Such mental state attribution can shape how viewers process unfolding narratives (e.g., Kopatich, Feller, Kurby, & Magliano, 2019; Levin, Hymel, & Baker, 2013; Magliano, Yan, Ackerman, Mccarthy, & Kurby, 2024; O'Neill & Shultis, 2007; Persson, 2003; Rooney & Bálint, 2018). In film, as in real life, this capacity draws on Spontaneous Theory of Mind (SToM)—the tendency to attribute beliefs, desires, and intentions without explicit prompting (Apperly, 2010; Kovács, Téglás, & Endress, 2010; Meins, Fernyhough, & Harris‐Waller, 2014). While some scholars distinguish between terms like Theory of Mind (ToM), mentalizing and mindreading (Quesque et al., 2024), we use SToM here as a broad term for mental state attribution that occurs intuitively and continuously in dynamic social contexts, including narrative media. Unlike traditional ToM tasks, which rely on explicit judgments (e.g., Wimmer & Perner, 1983), SToM is self‐initiated and continuously updated in response to contextual demands. This is particularly relevant for film viewing, where interpretation depends on viewers generating and revising mental state inferences as the narrative unfolds. The present study examines how such spontaneous belief attribution operates during naturalistic film viewing, focusing on its real‐time effects on cognitive processing and visual attention.

One narrative structure that systematically exploits asymmetries in what characters know is dramatic irony (DI), in which the audience possesses privileged information that is unavailable to one or more characters, creating a divergence in how events are interpreted (Lavandier, 2005). Beyond its narrative function, DI provides a context in which multiple, conflicting mental state representations must be spontaneously maintained and integrated to support ongoing narrative comprehension, making it a particularly informative framework for examining SToM in dynamic environments (Cabañas, Senju, & Smith, 2022). Although cinematic scenes are crafted stimuli, they offer rich, ecologically valid approximations of real‐world social understanding (Smith, 2012), as they embed SToM inferences within coherent narrative contexts. DI structurally parallels classic false belief paradigms, such as the Sally–Anne task (Baron‐Cohen, Leslie, & Frith, 1985; Wimmer & Perner, 1983), by clearly delineating what characters know versus what they ignore. In these scenes, viewers must spontaneously attribute false belief to the “victims” of irony—characters who act based on incomplete or inaccurate information. This makes DI a particularly suitable framework for examining how SToM shapes real‐time narrative processing, as it elicits unprompted mental state attribution while allowing belief asymmetries to be systematically manipulated. From a methodological perspective, this addresses a key limitation in existing ToM research: Traditional tasks offer experimental control but rely on discrete, prompted judgments, whereas more naturalistic approaches introduce contextual richness at the cost of experimental control (Achim et al., 2013; Apperly, 2012; Vanderwal et al., 2019).

To this end, the Dramatic Irony Film Corpus (Cabañas, Senju, & Smith, 2023) was developed, comprising a systematically constructed set of six edited scenes from classic Harold Lloyd silent comedies, structured into three narrative segments, informed by Lavandier (2005) and Cohn (2016): the *establisher*, introducing characters and their goals; the *installation*, presenting viewers with critical information unknown to certain characters; and the *exploitation*, where characters’ ignorance leads to observable consequences (see Fig. 1 for an illustrative example of these phases). Crucially, the experimental manipulation involved selectively presenting or omitting the installation segment, creating two conditions that differed only in viewers’ access to key narrative information. In the Installation condition, this segment provided viewers with privileged knowledge, enabling them to attribute a false belief to characters who lacked crucial information. In the Control condition, its omission ensured that viewers shared the characters’ limited perspective, producing a true‐belief scenario. By maintaining a strict correspondence in visual and temporal characteristics across both versions and varying only the narrative context— an approach supported by studies in event cognition and discourse processing (e.g., Magliano, Miller, & Zwaan, 2001; Rapp & Gerrig, 2006; Whitney et al., 2009)—this DI film corpus allows precise examination of how spontaneous false belief attribution influences viewers’ cognitive and attentional processing in real‐time scene comprehension. Using these movie segments and a free‐recall task, Cabañas et al. (2023) found that participants who viewed the installation segment formed more elaborate event models of characters’ mental states than those in the control condition. This elaboration reflected greater integration of the characters’ ignorance and misconceptions, leading to increased focus on cognitive aspects like beliefs and intentions over affective responses. This was reflected in higher mental state reference frequency, indicating deeper cognitive engagement in their interpretation of character actions.

**Fig. 1:**
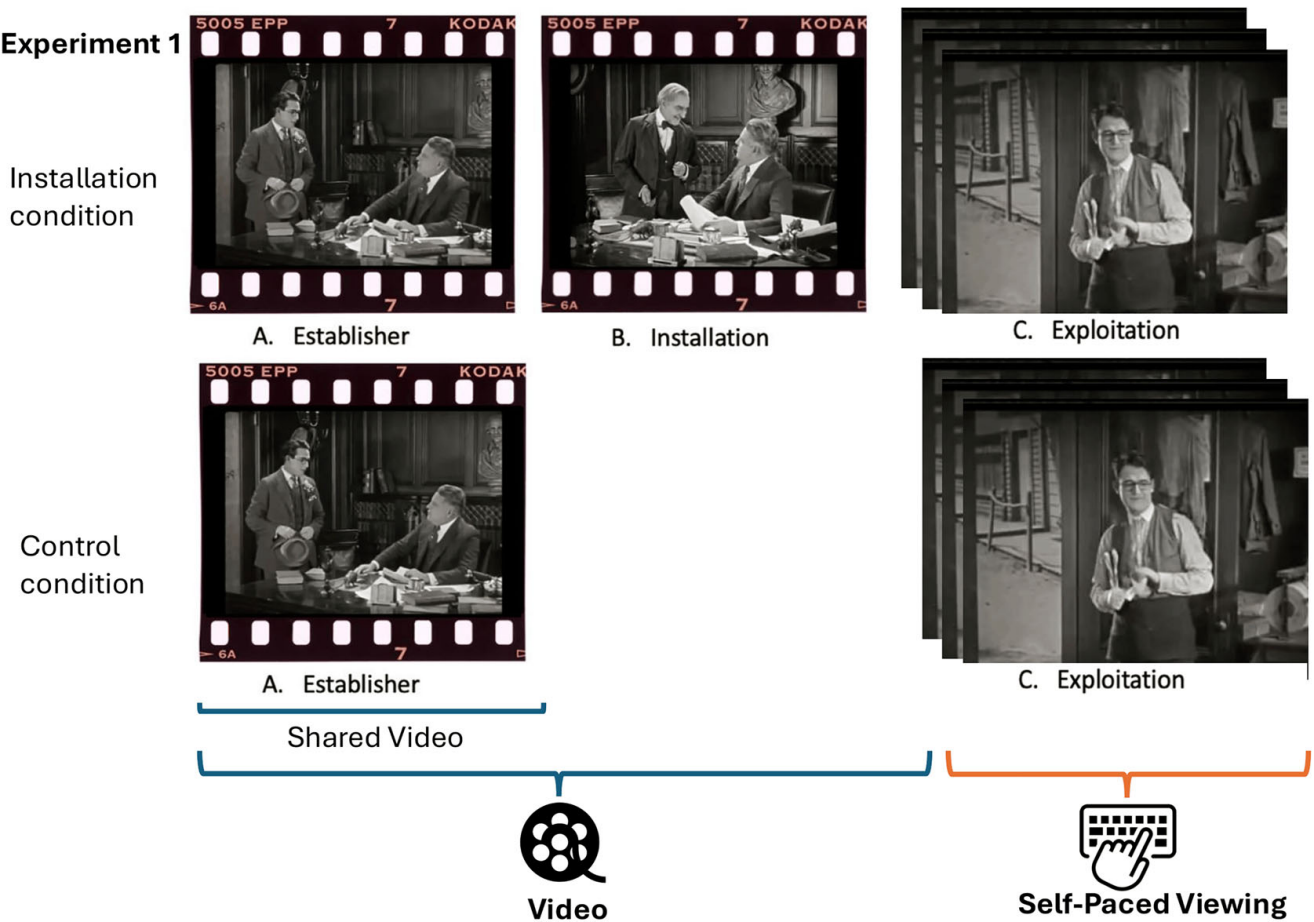
Experimental structure in Experiment 1 (Self‐Paced Viewing). *Note*. Example stills from Clip 4 (“Mistaken Rejection Letter”) from Girl Shy (Newmeyer & Taylor, 1924), illustrating the three narrative segments of a dramatic irony (DI) sequence—establisher (A), installation (B), and exploitation (C)—across conditions. In this clip, Harold receives a letter he believes to be a rejection but is in fact a cheque. In the Installation condition, viewers see all three segments, providing privileged information about the true content of the letter. In the Control condition, the installation segment is omitted, aligning viewer knowledge with the character's mistaken belief. The establisher segment was presented as a video (Shared Clip) across conditions. The exploitation segment was presented as a sequence of static frames under participant control (Self‐Paced Viewing). Stills courtesy of the Harold Lloyd Trust.

While these findings suggest that viewers spontaneously represent characters’ false beliefs during DI, they leave open the question of how these SToM processes unfold in real time. In particular, Cabañas et al. (2023) did not examine whether mental state reasoning is reflected in moment‐to‐moment processing differences, such as visual attention. Understanding how mental state reasoning guides visual attention is key to developing more accurate models of real‐time social cognition in naturalistic settings.

The present study builds on this by investigating whether SToM during DI manifests in viewers’ gaze behavior. Eye movements provide a time‐sensitive window into ongoing processing during scene viewing. While fixation location reflects what information is selected, fixation duration indexes how long that information is processed and is sensitive to both perceptual and cognitive demands (Henderson, 2003; Nuthmann, Smith, Engbert, & Henderson, 2010). Accordingly, variations in fixation duration offer a well‐established measure of moment‐to‐moment processing demands. Research on visual attention in static scenes has shown that gaze is actively guided by viewers’ internal representations and mental models (Castelhano, Mack, & Henderson, 2009; Henderson, 2003; Luke & Henderson, 2016), influencing fixation durations, saccade amplitudes, and object selection (Castelhano et al., 2009; Huettig, Rommers, & Meyer, 2011; Luke & Henderson, 2016). The Scene Perception and Event Comprehension Theory (SPECT; Loschky, Larson, Smith, & Magliano, 2020) similarly proposes that attention in dynamic scenes is guided by evolving mental models. Recent work on multimodal cohesion further shows that narrative structure and cross‐modal discourse cues (visual, verbal, and auditory) systematically guide attention and comprehension in film (Tseng et al., 2021). However, the extent to which these top‐down influences emerge during film viewing appears to depend on stimulus and task conditions. Smith and Mital (2013) demonstrated that a viewing task prioritizing static features of a dynamic scene (i.e., the background location) significantly altered gaze behavior, compared to free viewing. By comparison, Hutson, Smith, Magliano, and Loschky (2017) found minimal gaze differences despite comprehension variation, reinforcing the dominance of low‐level stimulus features, such as editing or camera movement—a phenomenon they refer to as “the tyranny of film.” But, similar to Smith and Mital (2013), when viewers were instructed to watch the scene in preparation for a map‐drawing task that competed with narrative comprehension, gaze became less synchronized and more exploratory (longer saccades, wider dispersion). Likewise, removing motion revealed comprehension‐driven gaze differences (Hutson, Chandran, Magliano, Smith, & Loschky, 2022), suggesting top‐down effects emerge when bottom‐up saliency is reduced. Further, research also suggests distinct visual attentional signatures depending on the type and availability of contextual knowledge. For instance, Pedziwiatr, Heer, Coutrot, Bex, and Mareschal (2023) found that prior event knowledge reduced visual exploration, while Liu, Li, Yeh, and Chien (2022) showed that higher cognitive load increased fixation durations, consistent with viewers dedicating more cognitive resources to the detailed processing of task‐relevant stimuli.

How these mechanisms unfold during SToM attribution in dynamic audiovisual scenes has been under‐researched. Although static ToM tasks such as the Director Task (Cane, Ferguson, & Apperly, 2017; Symeonidou, Dumontheil, Chow, & Breheny, 2016) have revealed perspective‐based gaze differences, they lack the contextual richness and ecological validity of narrative‐based social reasoning (Kingstone, Smilek, & Eastwood, 2008). This limits their ecological validity and their ability to capture the internally driven nature of SToM. To address these gaps, the current study examines whether spontaneous mentalizing processes modulate real‐time processing during scene comprehension.

A key challenge in real‐time narrative comprehension is the continuous updating of event models—especially when the narrative introduces information that conflicts with characters’ beliefs. Prior research has shown that this process is cognitively demanding, particularly when viewers must integrate new, incongruent information (e.g., Huff et al., 2018; Magliano & Zacks, 2011; Papenmeier, Boss, & Mahlke, 2019; Zacks & Swallow, 2007), including in the context of sequential images like comic strips (Cohn & Paczynski, 2013).

False belief scenarios like those present in DI exemplify this difficulty, as it requires viewers to simultaneously represent two conflicting realities: the actual state of the world and the character's mistaken belief about it (Csibra & Gergely, 1998, 2007; Perner, 1991). This dual‐representation process introduces conflicting belief representations and is assumed to place greater demands on cognitive resources than scenarios involving true beliefs, in which character knowledge aligns with reality and thus requires no such reconciliation. Studies using visual perspective‐taking tasks have consistently shown that judging another's mistaken belief increases reaction times (RTs) and error rates (Apperly, Riggs, Simpson, Chiavarino, & Samson, 2006; Keysar, Lin, & Barr, 2003; Samson, Apperly, Braithwaite, Andrews, & Bodley Scott, 2010), highlighting the cognitive demands of maintaining conflicting representations.

However, this evidence comes primarily from explicit ToM tasks, leaving open whether similar dynamics operate during spontaneous mentalizing in naturalistic contexts such as film viewing. Neuroimaging studies have begun to address this question by showing that belief‐relevant events engage Theory of Mind–related regions such as the temporoparietal junction (Levin et al., 2022; Raz et al., 2014; Richardson, Lisandrelli, Riobueno‐Naylor, & Saxe, 2018). Yet these studies typically examine responses to unfolding events without systematically manipulating the viewer's knowledge relative to the character. As a result, they identify when mentalizing is engaged but provide limited leverage on how belief information is integrated into event models over time. DI offers a structured case in which viewers and characters hold different beliefs about the same events, enabling direct comparison of identical scenes under distinct belief interpretations and isolating the contribution of belief information to ongoing comprehension.

The current study investigates how spontaneous false‐belief attribution during DI shapes real‐time cognitive processing and visual attention during film viewing across two experiments. Building on prior work showing that viewers retrospectively form richer mental models when exposed to DI (Cabañas et al., 2023), we examine whether these differences are also reflected during online scene comprehension, both in terms of cognitive effort and gaze allocation.

To this end, we combine eye‐tracking with a self‐paced viewing paradigm to compare viewers who possess privileged knowledge (Installation condition) with those who do not (Control condition), while holding the visual input constant. In this paradigm, participants view the exploitation segments as a sequence of static frames and advance at their own pace, allowing viewing times to index moment‐to‐moment processing demands (Cohn & Paczynski, 2013). Experiment 1 uses this frame‐by‐frame presentation to reduce the influence of bottom‐up cues such as motion, thereby increasing sensitivity to top‐down attentional processes. Experiment 2 assesses whether the same effects generalize to continuous film viewing, in which cinematic dynamics are preserved.

## 2 Experiment 1

Experiment 1 examines whether previously observed retrospective differences in comprehension (Cabañas et al., 2023) manifest as real‐time differences in cognitive effort during viewing. As a manipulation check, we predict that viewers in the Installation group, who receive narrative information unavailable to the character, will demonstrate greater understanding of the DI structure than viewers in the Control group, reflected in higher DI comprehension scores. This would indicate the construction of qualitatively different event models across conditions.

Hypothesis 1 concerns online cognitive effort. We predict that participants in the Installation group will exhibit greater processing demands than those in the Control group during exploitation scenes, reflecting the maintenance and use of false‐belief representations. In the Self‐Paced Viewing task, this should be observable as longer viewing durations (RTs) at specific moments where the discrepancy between the viewer's knowledge and the character's belief becomes relevant for interpretation. Because the visual content is largely shared across conditions, we do not predict a main effect of condition on overall RTs across scenes; instead, any differences are expected to emerge locally, at frames that require belief‐based reconciliation. Given that the clips vary in content and staging, we do not specify a priori the precise moments at which these effects will emerge and will instead identify these moments via a data‐driven exploratory approach. This approach is consistent with prior work using naturalistic stimuli, where cognitively relevant moments are identified through data‐driven analyses of continuous timecourses, including reverse‐correlation approaches that isolate event‐specific peaks in neural responses during movie viewing (e.g., Hasson et al., 2004; Richardson et al., 2018), as well as analogous approaches in behavioral measures such as eye‐tracking (e.g., Loschky, Larson, Magliano, & Smith, 2015).

We next examine whether spontaneous false‐belief attribution modulates visual attention across viewing phases. The Shared Clip refers to the establisher segment presented as a video clip, which is identical across conditions and provides a common baseline prior to the divergence in information access. The exploitation segment is then presented as a sequence of static frames under participant control (see Fig. 1). Based on prior work on context‐dependent attention in narrative comprehension, we advance two competing predictions. Hypothesis 2a predicts longer fixation durations in the Installation group during the frame‐by‐frame phase, consistent with increased cognitive load. Hypothesis 2b predicts a more exploratory viewing pattern, reflected in increased fixation count, larger saccade amplitudes, and greater gaze dispersion from screen center.

### 2.1 Method

#### 2.1.1 Design

Experiment 1 employed a mixed design, with one between‐subjects factor—condition (Installation vs. Control groups)—and one within‐subjects factor—phase (Shared Clip vs. Self‐Paced Viewing). The between‐subjects manipulation of narrative context (Installation vs. Control) was used to avoid drawing participants’ attention to the DI manipulation itself. We aimed to minimize the risk that exposing the same participants to both versions of a clip would make the knowledge asymmetry explicit, thereby encouraging more strategic or reflective processing rather than the spontaneous belief attribution under investigation. The primary dependent variables were: (1) *DI comprehension scores*, (2) *self‐paced RTs*, and (3) a series of eye‐tracking metrics indexing cognitive effort and attentional exploration. These included: *fixation duration* (reflecting processing load), *fixation count* (capturing the extent of information search), *saccade amplitude* (indicating visual exploration range), and *Euclidean distance between gaze position and screen center* (marking attentional deviation from default gaze position). Participants were randomly assigned to one of the two conditions, and the order of the six experimental clips was randomized for each participant.

#### 2.1.2 Participants

Thirty participants (13 female; *M* age = 30, *SD* = 9.24) were recruited via the university's SONA system and randomly assigned to either the Installation or Control group (*n* = 15 per group). An a priori power analysis using G*Power (Faul et al., 2009) determined a minimum sample of 24 participants to achieve 0.80 power, based on a large effect size (Cohen's *d* = 1.092) observed in Cabañas et al. (2023) for mental state reference differences. To account for potential exclusions, a 25% buffer was added, leading to a total of 30 recruited participants. Exclusion criteria included: prior familiarity with any film clips, insufficient English proficiency, diagnosis or ongoing assessment for autism spectrum conditions (due to documented differences in ToM processing compared with neurotypical populations; Chung, Barch, & Strube, 2014; Happé, 1994; Senju, 2012), and low‐effort responses (i.e., fewer than one sentence per scene in the free‐recall task). Based on these criteria, six participants were excluded: four due to technical issues or incomplete data, one for prior clip familiarity, and one for undergoing autism diagnosis. The final sample consisted of 24 participants (12 per group). Ethical approval was granted by the Birkbeck, University of London Ethics Board (ID: 181949). All participants provided written informed consent prior to participation.

#### 2.1.3 Stimuli

The study employed the Dramatic Irony Film Corpus (Cabañas et al., 2023), which features six excerpts from Harold Lloyd silent comedies (e.g., *Never Weaken*, 1921; *The Freshman*, 1925). Each excerpt was edited into two versions: a Control condition including only the establisher and exploitation scenes and an Installation condition, which additionally included the installation scene, thereby creating a knowledge disparity between viewer and character. Full details of the stimuli are provided in the corpus description (Cabañas et al., 2023) and Supporting Information.

All clips were silent, black‐and‐white and selected for their capacity to elicit spontaneous false belief attribution. This design ensured that both groups viewed visually and temporally matched clips that differed solely in narrative information. Clips ranged from 44 to 318 s, preceded by a 5‐s gray screen baseline to collect pupil data. Each clip was presented at 24 frames per second with a resolution of 720 × 480 pixels. Only the Exploitation scenes were shown in a self‐paced format. Slideshows were created by extracting every sixth frame (i.e., one frame per 250 ms) to approximate the natural pace of the original clip while minimizing participant fatigue. Resulting slideshows consisted of 156 to 247 frames per scene, depending on length. This extraction rate was informed by pilot testing, which showed that it preserved narrative continuity and allowed smooth, self‐paced progression. For a visual illustration of the DI structure and design, see Fig. 1; for detailed scene breakdowns, see Table 1 and Supporting Information Text S1. The corresponding clips are also available at the Open Science Framework (https://osf.io/by56s).

**Table 1:** Summary of film clips with respective Control and Installation versions, including their duration and a brief description of the scenes

Clip Title	Version	Duration	Short Description
Clip 1: Fake Injury Scheme	Control	2 m 21 s	Harold aims to showcase an osteopathic clinic's effectiveness by healing a man on the street, attracting new clients to the clinic where his love interest works.
	Installation	3 m 26 s	Harold devises a plan with an acrobat to stage a fake injury recovery, drawing the attention of potential clients to the osteopathic clinic where his love interest works.
Clip 2: False Team Membership	Control	3 m 26 s	Harold attempts to join the football team, successfully secures a spot and enthusiastically heads to the field to play.
	Installation	3 m 54 s	Harold excitedly tells a girl he made the team and eagerly goes to the field, not knowing that his real role is the water boy.
Clip 3: Jealous Misunderstanding	Control	1 m 9 s	Harold proposes to a girl and overhears a conversation where he finds out that she is being proposed to by another man.
	Installation	1 m 25 s	Harold proposes to a girl who accepts but later misinterprets her conversation with her brother, thinking she's being proposed to by another man.
Clip 4: Mistaken Rejection Letter	Control	2 m 26 s	Harold tries to publish his book but is rejected by the publisher and receives a rejection letter in the mail.
	Installation	3 m 6 s	Harold attempts to publish his book, and although initially rejected, the publisher reconsiders. Harold, believing the letter contains a rejection slip, tears up the unopened envelope containing a check.
Clip 5: Accidental Donation	Control	4 m 11 s	A missionary and his daughter write to Harold for help raising money for their mission. Harold comes across their mission cart and offers them a significant contribution.
	Installation	5 m 14 s	Harold accidentally burns a mission cart, writes a check to compensate, but is mistaken for a generous donor for the mission.
Clip 6: Mistaken Sheriff Identity	Control	1 m 32 s	Two men trick the sheriff into signing a permit for their traveling show.
	Installation	5 m 6 s	Harold, dressed as his father, the sheriff, is tricked into signing the permit for the two men.

#### 2.1.4 Apparatus

The experiment was run using Experiment Builder software (SR Research) on a 17‐inch CRT monitor with a resolution of 1024 × 768 pixels, viewed at a distance of 61 cm using a chin and forehead rest. This setup subtended a visual angle of 21° × 16°. Eye movements were recorded with an EyeLink 1000 desktop‐mounted eye tracker (SR Research, Version 1.5.2), sampling at 1000 Hz. Calibration used a nine‐point grid, with a maximum allowed spatial error of 1° of visual angle and an average error of 0.5° or less.

#### 2.1.5 Procedure

The study was conducted in person at the Birkbeck MERLiN lab in Central London (the United Kingdom). Upon arrival, participants were seated at a desk‐mounted eye‐tracker and completed a 45–60 min session. After calibration, participants viewed six film excerpts on a monitor (∼90 cm from chinrest), depending on their assigned group (Installation or Control). Each excerpt was preceded by a 5‐s gray screen (baseline). Participants first watched the establisher segment (and installation segment, if applicable) in full motion. Before the exploitation scene, a prompt in the top‐right corner indicated the beginning of a self‐paced slideshow. Participants advanced through the static frames by pressing the spacebar; reverse navigation was disabled, and no pacing instructions were given. RTs per frame were recorded.

After each clip, participants completed a free recall task to assess narrative comprehension, responding to the prompt: “What happened in the previous video and why?” allowing assessment of narrative comprehension without explicitly directing attention to mental states. DI comprehension was coded from these reports following the coding scheme in Cabañas et al. (2023), with inter‐rater reliability (25% sample) assessed using Krippendorff's alpha (α = 0.94). Coding was conducted by two coders blinded to condition using this predefined scheme. In cases of disagreement, coders discussed the instances until agreement was reached. Condition information was only introduced at the analysis stage, which was performed by a researcher not involved in coding.

After the six blocks, participants completed the Reading the Mind in the Eyes Test (RMET; Baron‐Cohen, Wheelwright, Hill, Raste, & Plumb, 2001) as a measure of ToM ability and answered open‐ended debriefing questions (e.g., “Did you notice anything similar across the clips?”). No participants reported awareness of the DI pattern. Participants were then fully debriefed.

### 2.2 Results

#### 2.2.1 Data cleaning and analysis

The analysis plan for the self‐paced RTs was prespecified in a preregistration document that can be found on the Open Science Framework (https://osf.io/by56s). RMET scores were excluded from the main analyses because the task was administered after the eye‐tracking session, which limits their interpretability as baseline measures of mentalizing ability. This ordering was chosen to avoid priming participants to focus on mental state reasoning prior to the viewing task, thereby preserving the naturalistic, unprompted nature of spontaneous mentalizing. However, prior work suggests that engagement with narrative film can transiently influence ToM performance, likely by inducing a shift toward mentalizing‐based processing during interpretation (Castano, 2021; Black & Barnes, 2015), introducing ambiguity as to whether scores reflect stable individual differences or task‐induced variation. Accordingly, RMET scores were not used for analytical purposes and are reported only descriptively to characterize the sample. No significant difference between conditions was observed, *t*(22) = −1.51, *p* = .145, 95% CI [−3.75, 0.59], *d* = 0.62.

To ensure valid responses, we first discarded self‐paced RTs above 2000 ms. This initial cutoff was informed by task parameters: Since each frame was designed to require rapid sequential viewing, excessively long RTs likely reflected momentary disengagement or distraction rather than true cognitive processing. Thus, the 2000 ms threshold functioned as a safeguard against values incompatible with the task's real‐time demands. Subsequently, we computed the mean and standard deviation of the remaining RTs and applied a 3SD trimming procedure. This is a standard approach to remove statistical outliers while preserving the majority of the data. We opted for a 3SD (rather than 2SD) criterion to avoid overly aggressive exclusion, thus retaining potentially informative responses at the longer end of the distribution. All data handling and statistical analyses were conducted in R using RStudio.

#### 2.2.2 Manipulation check: DI comprehension scores

To verify the effectiveness of the experimental manipulation, we compared participants’ DI comprehension scores between the Installation and Control groups. A Welch two‐sample *t*‐test revealed significantly higher DI comprehension scores in the Installation group (*M* = 8.75, *SD* = 2.30), compared to the Control group (*M* = 0.83, *SD* = 1.03), *t*(15.24) = −10.88, *p* < .001, *d* = 4.44.

We also analyzed DI comprehension scores for each of the six clips separately. Welch's *t*‐tests with Bonferroni correction showed significant differences across all clips: Clips 1, 5, and 6 showed moderate effects (all *p* < .006), while Clips 2, 3, and 4 exhibited stronger differences (*p* < .0001). These per‐clip results can be visualized in Fig. 2.

**Fig. 2:**
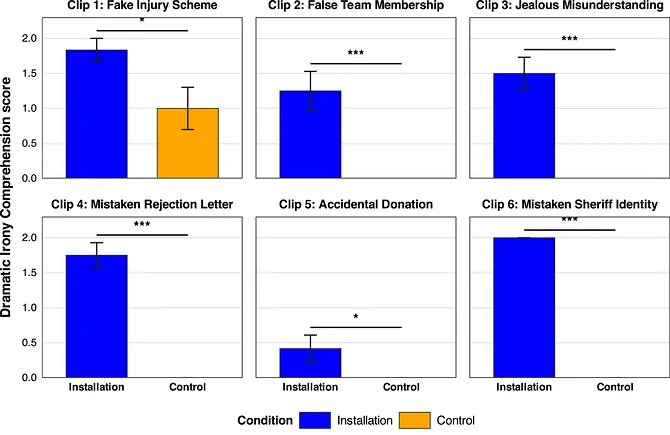
Bar plot depicting DI comprehension scores by condition and clip (Installation vs. Control) in Experiment 1. *Note*. Error bars represent standard errors of the mean.

### 2.3 Self‐paced RTs

#### 2.3.1 Overall RT analysis across all clips

To assess whether spontaneous false belief attribution increases cognitive effort, we analyzed self‐paced RTs during the exploitation scenes—identical across conditions but preceded by differing narrative contexts. Descriptive statistics for self‐paced RTs are shown in Table 2, indicating that participants typically spent approximately 390–400 ms per frame; for a breakdown by clip and condition, see Supporting Information Table S1. A linear mixed effects (LME) model approach was used on log‐transformed RTs to address data skewness and account for both participant‐level and clip‐level variability. Frame was operationalized as a within‐clip index. Given the high number of repeated observations per clip, temporal dependence was modeled using an autoregressive error structure (AR1). A random‐effects structure was determined through stepwise model comparison. Attempts to include random slopes (Barr, Levy, Scheepers, & Tily, 2013) resulted in convergence issues and singular fits, likely reflecting overparameterization given the nested structure of the data and sample size. We therefore adopted a parsimonious model with random intercepts for participants and clips. This approach follows recommendations to balance model complexity and statistical power (Matuschek, Kliegl, Vasishth, Baayen, & Bates, 2017).

**Table 2:** Mean self‐paced reaction times (RTs; ms) per frame by condition

Condition	Mean RT (ms)	*SD*
Control	389	177
Installation	398	198

Stepwise model comparison indicated that including random intercepts for clips substantially improved model fit, χ^2^(1) = 985.86, *p* < .001, whereas adding condition alone did not, χ^2^(1) = 0.73, *p* = .393. Adding frame improved fit, χ^2^(1) = 284.05, *p* < .001, reflecting overall temporal variation in RTs across clips. The global condition × frame interaction was not significant, χ^2^(1) = 1.70, *p* = .192. However, a model including the condition × clip interaction significantly improved fit relative to the condition‐only model, χ^2^(10) = 199.89, *p* < .001, indicating that condition effects varied across clips. We therefore proceeded with clip‐specific analyses to identify where RT differences emerged. Full model outputs are reported in the Supporting Information (Tables S2–S4).

#### 2.3.2 Clip‐specific RT analysis

To locate where effects occurred, separate LMEs were conducted for each clip. Consistent with Hypothesis 1, Clip 1 (“Fake Injury Scheme”), Clip 2 (“False Team Membership”), Clip 4 (“Mistaken Rejection Letter”), and Clip 6 (“Mistaken Sheriff Identity”) showed significantly longer RTs for the Installation group at specific frames (*p* < .05 for the condition × frame interaction), indicating greater cognitive effort. No significant effects were found for Clip 3 (“Jealous Misunderstanding”) or Clip 5 (“Accidental Donation”). Given the poor DI comprehension observed for Clip 5 (“Accidental Donation”; mean < 1), it was excluded from further analysis. To ensure that this decision did not influence the results, we conducted a sensitivity analysis excluding this clip. The pattern of model comparisons and fixed effects remained unchanged, indicating that the reported results were not driven by its inclusion.

For Clip 1 (“Fake Injury Scheme”), Clip 2 (“False Team Membership”), Clip 4 (“Mistaken Rejection Letter”), and Clip 6 (“Mistaken Sheriff Identity”), we identified specific frames with significantly higher RTs in the Installation group using Benjamini–Hochberg‐corrected contrasts. These frame‐level effects are shown in Fig. 3, with detailed statistics reported in Tables S5–S9.

**Fig. 3:**
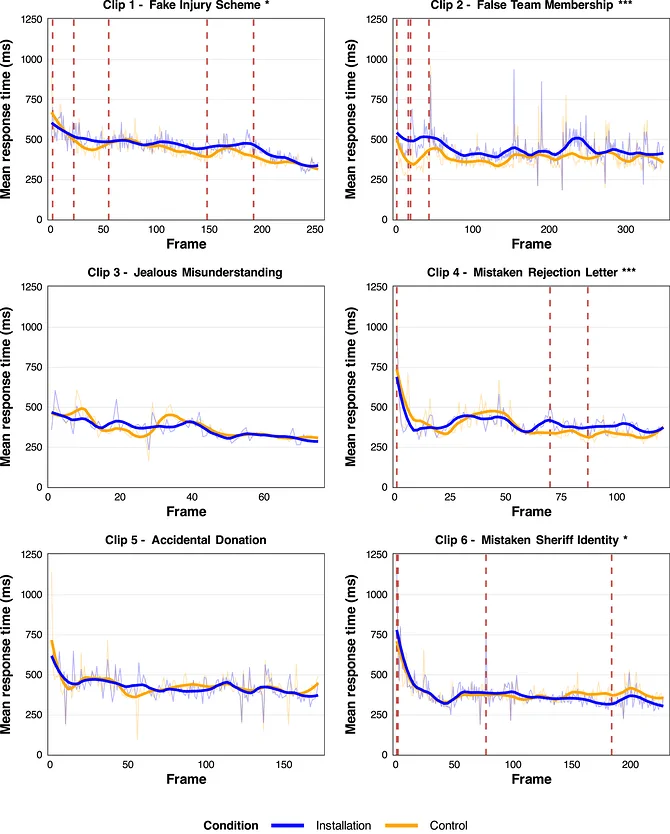
Self‐paced reaction times (RTs) across frames and conditions per clip. *Note*. Asterisks in clip titles denote clips for which the condition × frame interaction was significant in the clip‐level model (^*^ *p* < .05, ^**^ *p* < .01, ^***^ *p* < .001). For each clip, the plot shows mean self‐paced RTs across frames for the Control and Installation conditions. Red vertical lines mark frames at which the condition contrast reached statistical significance based on clip‐specific linear mixed effects models with Benjamini–Hochberg‐corrected comparisons (*p* < .05).

#### 2.3.3 Eye‐tracking metrics under Self‐Paced Viewing (vs. shared video)

To test whether prior exposure to the installation scene altered visual attention patterns during shared exploitation scenes, we analyzed four eye‐tracking measures using mixed two‐way ANOVAs: Fixation Duration, Fixation Count, Saccade Amplitude, and Distance to Screen Center. Each analysis included phase (Shared Clip vs. Self‐Paced Exploitation) as a within‐subjects factor and condition (Installation vs. Control) as a between‐subjects factor, with results summarized in Fig. 4.

**Fig. 4:**
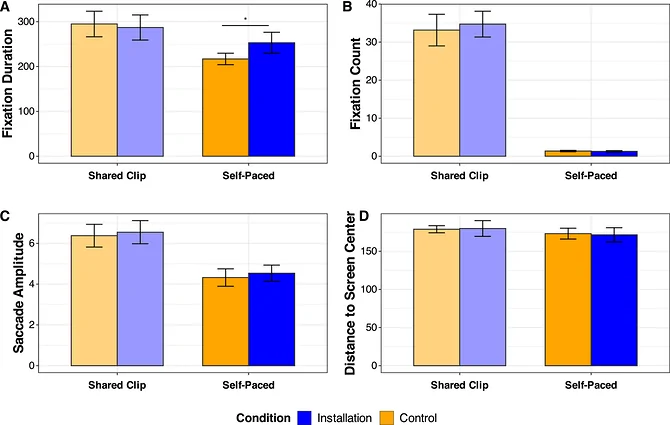
Comparative visualization of eye‐tracking metrics across different viewing conditions in Experiment 1. *Note*. This figure comprises four subplots (A–D) each representing a distinct eye‐tracking metric: (A) Fixation Duration, (B) Fixation Count, (C) Saccadic Amplitude and (D) Distance from Center. Error bars represent standard errors of the mean. Significance asterisks (*) indicate statistically significant differences between conditions in the phase of interest (self‐paced viewing phase; * = *p* < .05, ** = *p* < .01, and *** = *p* < .001).

#### 2.3.4 Fixation duration

A significant main effect of phase was found (*F*(1, 22) = 59.29, *p* < .001, η^2^ = 0.37), alongside a significant phase × condition interaction (*F*(1, 22) = 9.17, *p* = .006, η^2^ = 0.09). As predicted by Hypothesis 2a, planned contrasts revealed longer fixation durations in the Installation group during the Self‐Paced Viewing phase (*M* = 253.13 ms) than in the Control group (*M* = 216.95 ms), *t* = 2.34, *p* = .025, *d* = 0.41.

#### 2.3.5 Fixation count

Fixation count violated normality assumptions (Shapiro–Wilk *p* < .001), so we applied the aligned rank transform. Neither main effects nor interaction effects were significant (all *p* > .5), providing no support for Hypothesis 2b, which predicted increased visual exploration in the Installation condition.

#### 2.3.6 Saccade amplitude

A main effect of phase was observed (*F*(1, 22) = 227.92, *p* < .001, η^2^ = 0.65); saccade amplitudes were longer in the shared establisher clip than the self‐paced exploitation clip, but no effects of condition or interaction emerged (*p* > .5), providing no support for Hypothesis 2b.

#### 2.3.7 Distance to screen center

Similarly, a main effect of phase was significant (*F*(1, 22) = 15.98, *p* < .001, η^2^ = 0.08), with greater scene exploration in the shared establisher clip than the self‐paced exploitation clip, but there were no significant condition or interaction effects (*p* > .47), again providing no support for Hypothesis 2b.

#### 2.3.8 Exploratory illustration of gaze allocation during Self‐Paced Viewing

To complement the confirmatory analyses, we employed a data‐driven exploratory approach, following Haensel et al. (2020) and Võ, Smith, Mital, and Henderson (2012), to visualize gaze patterns using heat maps and to conduct a focused qualitative examination of gaze allocation to narratively critical objects. We focus on a single illustrative case (Clip 4, “Mistaken Rejection Letter”), in which the false‐belief conflict hinges on a clearly identifiable object. In this clip, Harold attempts to publish his book and appears to receive a rejection, although the publisher has in fact sent a cheque, of which Harold is unaware.

The analysis was restricted to two frames (Frames 70 and 87) that showed significant condition differences in the preceding RT contrasts, allowing us to examine whether privileged knowledge was associated with differential allocation of attention to belief‐relevant elements. In both frames, viewers in the Installation condition showed greater attention to the letter relative to Harold's face, compared to viewers in the Control condition (see Fig. 5). Analogous visualizations for the remaining clips are provided in the Supporting Information (Figs. S7–S9).

**Fig. 5:**
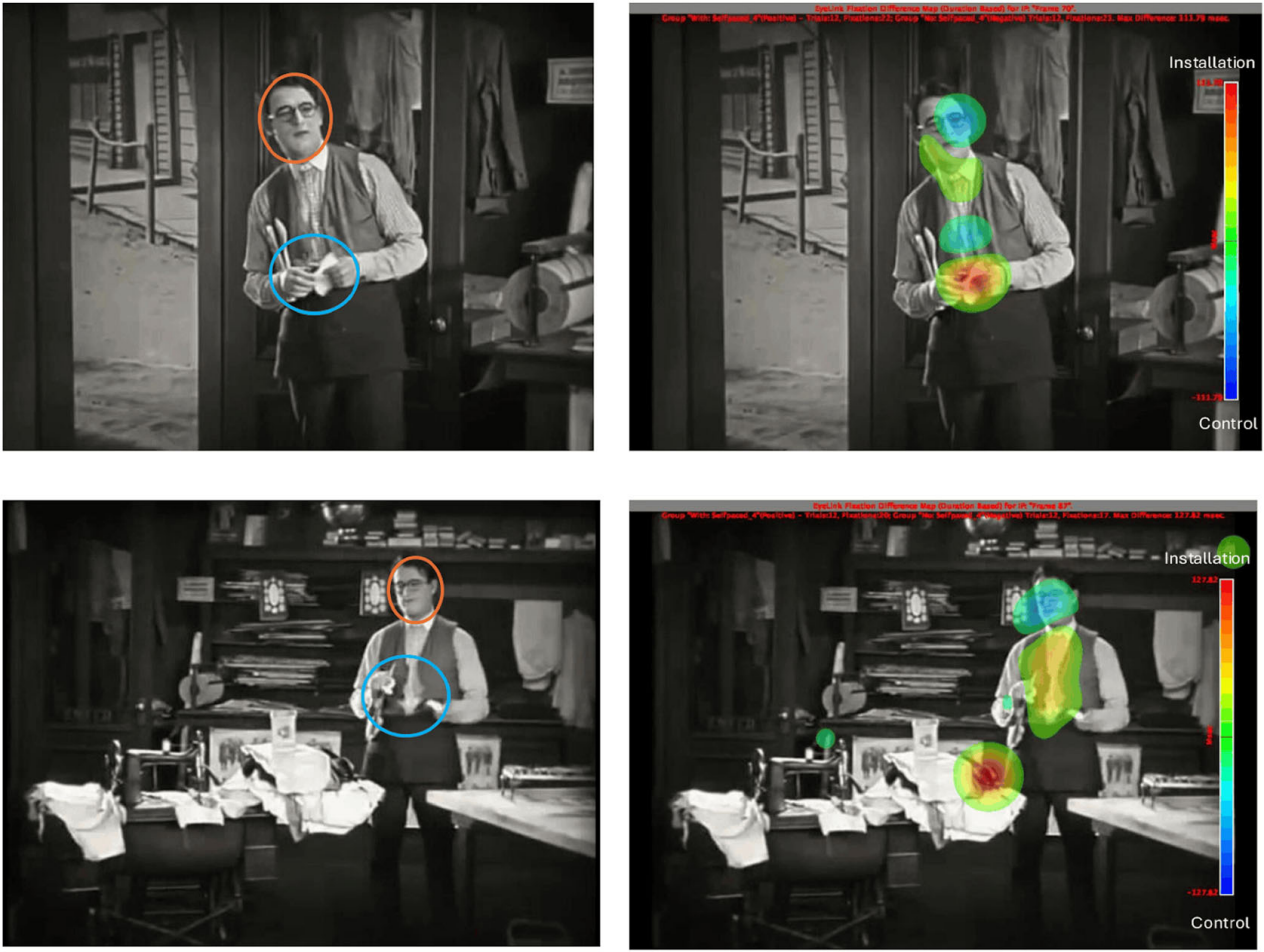
Narratively critical elements and between‐condition fixation static heat maps for Clip 4 (“Mistaken Rejection Letter”). *Note*. The right panel shows narratively critical elements. The left panel displays static heat maps showing fixation duration differences between the Installation and Control groups for Frame 70 (top) and Frame 87 (bottom). Warmer colors indicate longer fixations in the Installation group relative to the Control group; cooler colors indicate longer fixations in the Control group relative to the Installation group.

#### 2.3.9 Rationale for Experiment 2

The pattern of results observed in Experiment 1, together with the exploratory analyses (Fig. 5; Supporting Information Figs. S7–S9), indicates that spontaneous false‐belief attribution modulates moment‐to‐moment processing under reduced‐motion conditions (i.e., self‐paced slideshow viewing). Effects on gaze varied across clips, suggesting sensitivity to scene composition, which may account for the absence of effects in some cases (e.g., Clip 3 “Jealous Misunderstanding”). At the same time, oculomotor measures consistently indicated increased processing demands in the Installation condition during the Exploitation phase, as reflected in longer fixation durations.

An open question is whether these effects generalize to video viewing, where gaze is strongly constrained by motion and editing. Such constraints often limit the expression of comprehension‐driven differences, a phenomenon referred to as the “tyranny of film” (Loschky et al., 2015). Prior work shows that gaze in video is largely dominated by bottom‐up features unless viewing conditions are modified, for example, through explicit task demands that redirect attention (Hutson et al., 2017) or by reducing motion (Hutson et al., 2022). Examining these effects during video viewing therefore provides a stringent test of their robustness under more natural viewing conditions.

Experiment 2 addresses this question by preserving the manipulation of narrative information while removing the frame‐by‐frame presentation. Accordingly, Hypothesis 3 follows the logic of Experiment 1, with condition differences expected to emerge specifically during the Exploitation segment, rather than the Establisher segment, in which viewers share the same knowledge base. Hypothesis 3a predicts longer fixation durations in the Installation condition, reflecting increased processing demands during belief‐based interpretation. Hypothesis 3b predicts a shift in visual sampling, reflected in changes in fixation rate, saccade amplitude and gaze dispersion from screen center.

## 3 Experiment 2

### 3.1 Methods

#### 3.1.1 Design

In Experiment 2, narrative context was manipulated within participants using a clip‐level counterbalancing scheme. Each participant viewed clips from both conditions (Installation vs. No Installation), with stimuli distributed across lists such that each clip appeared in both conditions across participants. The two groups implement complementary list structures: Participants in Group 1 viewed two Installation and three No Installation clips, whereas Group 2 received the inverse. Across participants, this ensures that each clip appears equally often in both conditions. This yields a crossed repeated‐measures design, with observations nested within both participants and stimuli. The full counterbalancing scheme is provided in the Supporting Information Table S10.

Relative to Experiment 1, all film segments were presented as videos and the manipulation was implemented within participants, allowing direct comparison of attentional responses across conditions within the same individual under more naturalistic viewing conditions. This approach reduces between‐participant variability while ensuring that effects of the manipulation are not confounded with stimulus‐specific properties.

Dependent variables were restricted to eye‐tracking indices of cognitive load and attentional exploration during video viewing, including fixation duration, fixation rate, saccade amplitude, and Euclidean distance to screen center. Fixation rate was used instead of fixation count because segment durations varied across continuous film clips, allowing comparison of visual sampling while accounting for differences in viewing time and enabling direct comparison across film segments (Establisher vs. Exploitation).

#### 3.1.2 Participants

A total of 87 participants completed the in‐lab eye‐tracking session. Inclusion criteria for the in‐lab component consisted of normal or corrected‐to‐normal vision, an advanced level of English proficiency (self‐reported), no prior diagnosis of a neurodevelopmental condition (e.g., ASD, ADHD, dyslexia), and sufficient data quality. Of the 87 participants, 22 were excluded: 10 due to missing or unusable eye‐tracking data, one due to low engagement—operationalized as providing only minimal (one‐sentence) descriptions in the free‐recall task despite instructions to write a paragraph—six due to insufficient English proficiency, and five due to voluntary disclosure of a neurodevelopmental diagnosis (four ADHD, one dyslexia). The final analytic sample therefore consisted of 65 participants (32 female; *M* age = 30.49 years, *SD* = 10.83), all of whom reported normal or corrected‐to‐normal vision.

The target sample size was based on a repeated‐measures approximation using the phase × condition interaction observed for fixation duration in Experiment 1 (*η* ^2^ = 0.09; *f* ≈ 0.31), which indicated that approximately 84 participants would be required to achieve 80% power at α = 0.05. However, Experiment 2 employed a within‐subjects, crossed design in which each participant contributed observations across conditions and stimuli, increasing statistical sensitivity relative to the between‐subjects design used in Experiment 1 (Westfall, Kenny, & Judd, 2014). Ethical approval was granted by the university ethics committee. All participants provided written informed consent prior to participation.

#### 3.1.3 Stimuli

Stimuli consisted of five silent film clips drawn from the Dramatic Irony Film Corpus (Cabañas et al., 2023); Clip 5 (“Accidental Donation”) was omitted due to consistently low comprehension scores and the absence of reliable effects in Experiment 1. Each clip comprised three narrative segments (Establisher, Installation, Exploitation), with the same manipulation of narrative information as in Experiment 1 (i.e., inclusion vs. omission of the installation segment; see Fig. 6).

**Fig. 6:**
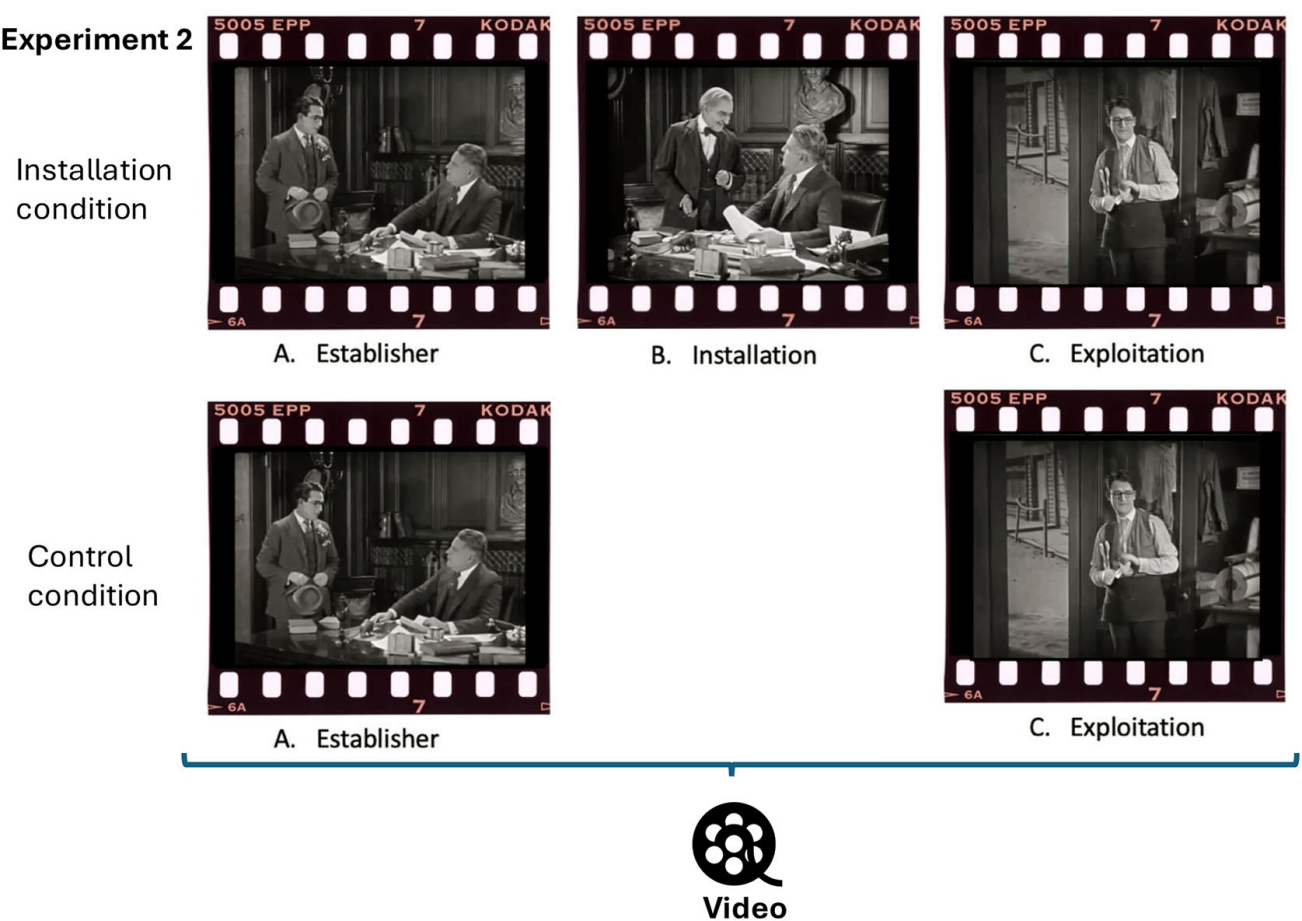
Experimental structure in Experiment 2 (video viewing). *Note*. Example stills from Clip 4 (“Mistaken Rejection Letter”) from Girl Shy (Newmeyer & Taylor, 1924), showing the three segments across conditions. As in Fig. 1, the Installation condition includes the installation segment, whereas the Control condition omits it; all segments were presented as continuous video. Stills courtesy of the Harold Lloyd Trust.

#### 3.1.4 Apparatus

Apparatus was identical to that used in Experiment 1 (EyeLink 1000, 1000 Hz sampling; same display and calibration parameters).

#### 3.1.5 Procedure

The procedure of the in‐lab component of this experiment followed that of Experiment 1 unless otherwise noted. Participants completed a 90‐min in‐lab session with standard eye‐tracking calibration. All clips were presented as videos, without a self‐paced viewing component. Participants viewed each excerpt in full while eye movements were recorded. After each clip, they completed a free‐recall task (“What happened in the previous video and why?”). DI comprehension was coded from these responses using the same blinded coding procedure as in Experiment 1 (Krippendorff's α = 0.89). Following the task, participants were asked whether they noticed any recurring pattern; none reported awareness of the manipulation. Additional cognitive measures (e.g., WASI‐II subscales and working memory span tasks) were administered after the main task as part of a broader project but are not analyzed here.

### 3.2 Results

#### 3.2.1 Manipulation check: DI comprehension

To verify the effectiveness of the manipulation under the within‐participants design, DI comprehension scores were analyzed using a LME model of the form:

DI Comprehension ∼ condition + (1 | participant) + (1 | clip),

With condition (Installation vs. Control) as a fixed effect and random intercepts for participants and clips. This approach accounts for the non‐independence of observations arising from the crossed structure of the data (Baayen, Davidson, & Bates, 2008). Given the absence of frame‐level temporal structure and the confirmatory nature of the analysis, the random‐effects structure was specified a priori and the final model is reported without stepwise comparison. This differs from Experiment 1, where model comparison was necessary due to the inclusion of frame‐level predictors and convergence constraints.

The analysis revealed a robust effect of condition, with higher comprehension scores in the Installation condition relative to the Control condition (β = 0.96, *SE* = 0.04, *t* = 24.98, *p* < .001, 95% CI [0.89, 1.04]). This indicates that the manipulation successfully induced privileged knowledge across clips even when both conditions were experienced within participants.

At the clip level, condition effects were examined separately using linear models. These analyses showed that the Installation condition consistently yielded higher DI comprehension across clips, confirming that the manipulation generalized across stimuli. Visual inspection of Fig. 7 indicates some variability in effect magnitude across clips, while the direction of the effect remained consistent.

**Fig. 7:**
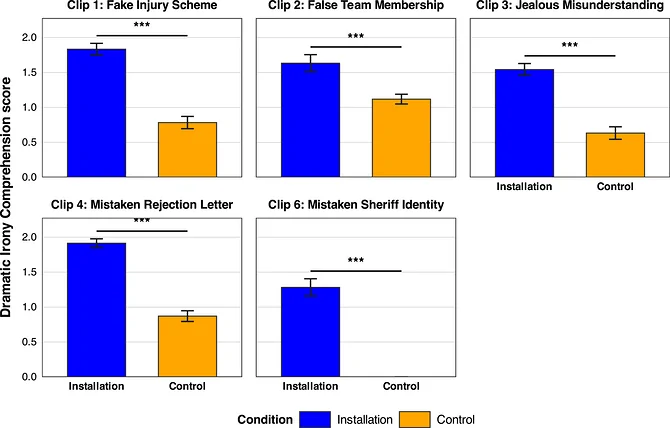
Bar plot depicting DI comprehension scores by condition and clip (Installation vs. Control) in Experiment 2 (within‐participants design). *Note*: Error bars represent standard errors of the mean.

#### 3.2.2 Eye‐tracking metrics during video viewing

To assess whether prior exposure to the installation scene modulated visual attention during video viewing, eye‐tracking measures were analyzed using LME models with fixed effects of condition (Installation vs. Control) and phase (Establisher vs. Exploitation) and random intercepts for participants and stimuli. Given that Hypothesis 3 a priori specified condition differences in the Exploitation phase, planned comparisons between conditions were conducted within each phase regardless of the presence of a significant interaction. Results for all eye‐tracking measures are shown in Fig. 8.

**Fig. 8:**
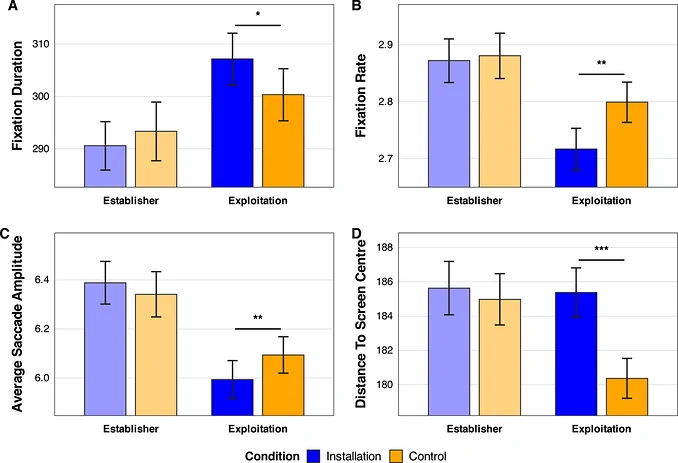
Comparative visualization of eye‐tracking metrics across video‐viewing conditions in Experiment 2. *Note*. The figure comprises four subplots (A–D), each representing a distinct eye‐tracking metric: (A) fixation duration, (B) fixation rate, (C) saccade amplitude, and (D) distance to screen center. Error bars represent standard errors of the mean. Significance asterisks (*) indicate statistically significant simple effects within phase (* = *p* < .05, ** = *p* < .01, and *** = *p* < .001).

#### 3.2.3 Fixation duration

No main effects of phase (β = 7.24, *SE* = 4.47, *t*(698) = 1.62, *p* = .106, 95% CI [−1.54, 16.01]) or condition (β = −2.66, *SE* = 4.44, *t*(702) = −0.60, *p* = .549, 95% CI [−11.36, 6.04]) were observed. A significant phase × condition interaction emerged (β = 14.10, *SE* = 6.38, t(699) = 2.21, *p* = .028, 95% CI [1.59, 26.60]). Consistent with the a priori prediction of Hypothesis 3a, planned comparisons showed longer fixation durations in the Installation condition during the Exploitation phase (β = 11.44, *SE* = 4.65, *t*(700) = 2.46, *p* = .014, 95% CI [2.32, 20.55]), with no difference in the Establisher phase (β = −2.66, *SE* = 4.44, *t*(702) = −0.60, *p* = .549, 95% CI [−11.36, 6.04]).

#### 3.2.4 Fixation rate

Fixation rate was computed as the number of fixations per second within each segment. Fixation rates were lower during the Exploitation phase than during the Establisher phase (β = −0.08, *SE* = 0.03, *t*(702) = −2.48, *p* = .014, 95% CI [−0.14, −0.02]). The main effect of condition was not significant (β = −0.05, SE = 0.03, *t*(705) = −1.68, *p* = .094, 95% CI [−0.12, 0.01]), nor was the interaction (β = −0.08, *SE* = 0.05, *t*(703) = −1.77, *p* = .077, 95% CI [−0.17, 0.01]). Consistent with the directional prediction of Hypothesis 3b, planned comparisons indicated lower fixation rates in the Installation condition during the Exploitation phase (β = −0.14, *SE* = 0.03, *t*(703) = −4.05, *p* < .001, 95% CI [−0.21, −0.07]), whereas no difference was observed during the Establisher phase (β = −0.05, SE = 0.03, *t*(705) = −1.68, *p* = .094, 95% CI [−0.12, 0.01]).

#### 3.2.5 Saccade amplitude

Saccade amplitudes were reduced during the Exploitation phase relative to the Establisher phase (β = −0.27, *SE* = 0.06, *t*(698) = −4.45, *p* < .001, 95% CI [−0.39, −0.15]). There was no main effect of condition (β = 0.00, *SE* = 0.06, *t*(701) = 0.04, *p* = .969, 95% CI [−0.12, 0.12]), but the interaction was significant (β = −0.19, *SE* = 0.09, *t*(699) = −2.13, *p* = .033, 95% CI [−0.36, −0.01]). As specified in Hypothesis 3b, planned comparisons indicated lower saccade amplitudes in the Installation condition during the Exploitation phase (β = −0.18, *SE* = 0.06, t(700) = −2.90, *p* = .004, 95% CI [−0.31, −0.06]), with no difference in the Establisher phase (*p* = .969, 95% CI [−0.12, 0.12]).

#### 3.2.6 Distance to screen center

Gaze was more centrally focused during the Exploitation phase (β = −4.61, *SE* = 1.54, *t*(698) = −3.00, *p* = .003, 95% CI [−7.62, −1.60]). The main effect of condition was not significant (β = 1.07, *SE* = 1.55, t(703) = 0.69, *p* = .492, 95% CI [−1.97, 4.10]), but the interaction was significant (β = 4.36, *SE* = 2.17, t(698) = 2.01, *p* = .045, 95% CI [0.11, 8.61]).

Partially consistent with Hypothesis 3b, planned comparisons showed greater distance from center in the Installation condition during the Exploitation phase (β = 5.42, *SE* = 1.55, *t*(703) = 3.50, *p* < .001, 95% CI [2.39, 8.45]), with no difference in the Establisher phase (*p* = .492, 95% CI [−1.97, 4.10]).

#### 3.2.7 Exploratory illustration of gaze allocation during continuous viewing

We extended the exploratory visualization from Experiment 1 to the continuous‐viewing task by generating dynamic gaze heat maps centered on the visual moments corresponding to the diagnostic frames identified in Experiment 1. For each diagnostic frame, we identified the equivalent visual moment in the continuous videos and extracted a 1‐s window of gaze data (±12 video frames).

As in Experiment 1, we focus on the same example (Clip 4, *Mistaken Rejection Letter*). However, unlike the static heat maps in Experiment 1, which represented gaze for individual diagnostic frames accumulated over a viewing period of ∼400 ms, the dynamic heat maps summarize gaze frame‐by‐frame (41.2 ms) and are accordingly much sparser and susceptible to noise due to individual differences. Across both diagnostic moments, viewers in both conditions spent most of the viewing periods dividing their attention between the letter and Harold's face, but participants in the Installation condition returned more frequently to the letter, consistent with the qualitative pattern observed in the static slideshow version (Fig. 9). Similar patterns between the dynamic (E2) and static slideshow versions of the scenes (E1) are visible in the remaining clips (Figs. S10–S14).

**Fig. 9:**
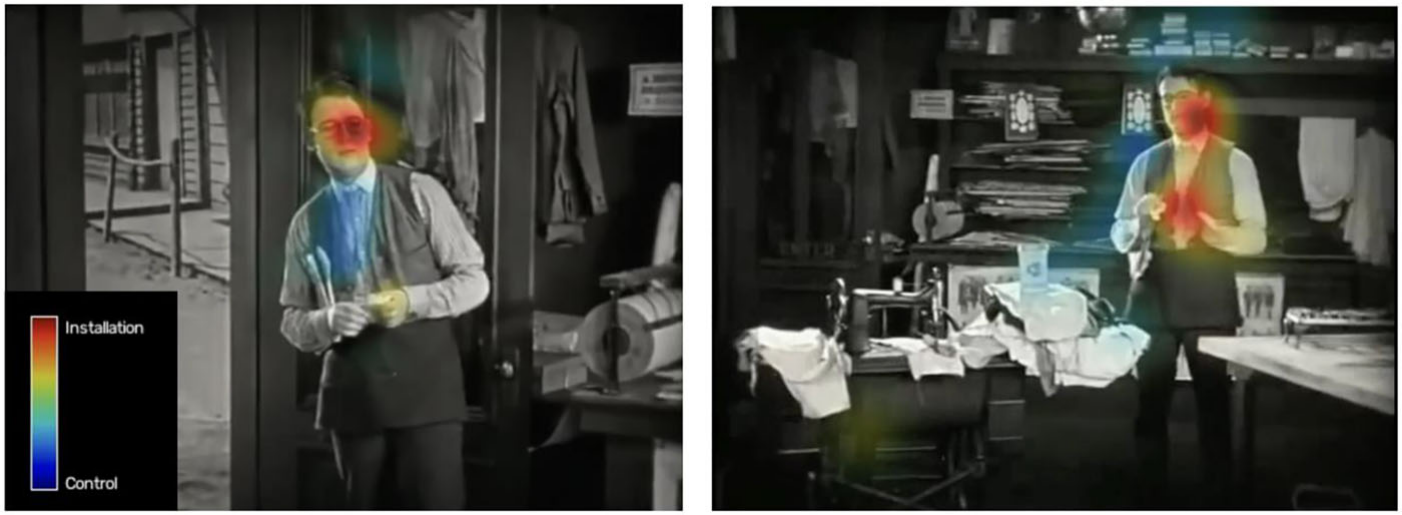
Dynamic Fixation Heat Maps for Clip 4 (“Mistaken Rejection Letter”). Note. Dynamic heat maps illustrate gaze allocation during the video frame (41.2 ms) centered on the visual moments corresponding to Frames 70 (left) and 87 (right) from Experiment 1. For each event, warmer colors indicate greater gaze concentration at that part of the image across participants in the Installation group, whereas cooler colors indicate more gaze in the Control group.

**Fig. 10:**
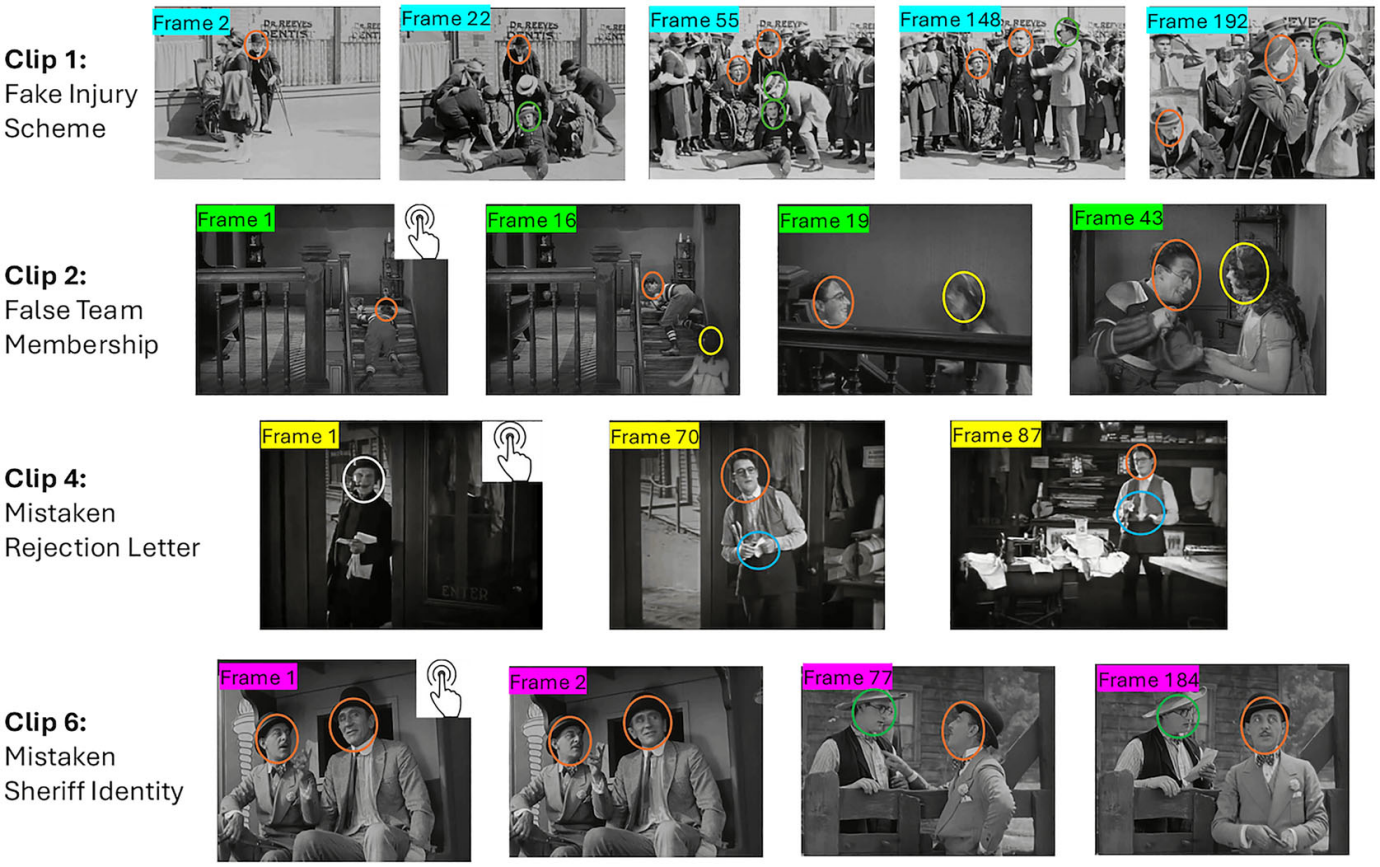
Frames showing significant condition differences in self‐paced RTs during the Exploitation phase. *Note*. Frames depict time points within the exploitation phase that showed significant condition differences in frame‐level self‐paced RT analyses (see Fig. 3 and Table S11). Colored circles indicate narratively relevant elements: Orange marks targets of the DI (characters holding the false belief), green indicates the protagonist, yellow denotes observing or interacting characters, blue highlights objects central to the unfolding event, and white outlines background characters.

To aid interpretation of the difference heatmaps, the individual condition heat maps are presented alongside the corresponding difference maps to provide context for the observed differences (Supporting Information Figs. S12 and S13).

### 3.3 Discussion

#### 3.3.1 Summary of findings across experiments

The present study examined whether exposure to false‐belief‐inducing DI clips elicits distinct patterns of cognitive processing, compared to matched true‐belief control clips and whether patterns of visual attention reflect the construction and maintenance of SToM representations during real‐time comprehension. Across two studies, DI elicited greater cognitive effort and systematic shifts in visual attention. Participants exposed to the Installation condition showed longer fixation durations, indicating increased processing demands associated with spontaneous false‐belief attribution during ongoing scene interpretation. To our knowledge, this is the first study to demonstrate this effect using naturalistic yet experimentally controlled stimuli.

Consistent with prior findings (Cabañas et al., 2023), participants in both experiments demonstrated significantly greater comprehension of DI scenarios, confirming that the manipulation induced qualitatively different event models. Clip 5 (“Accidental Donation”) yielded the lowest comprehension scores—below partial understanding—and was therefore excluded from subsequent analyses and omitted from Experiment 2.

#### 3.3.2 Experiment 1: Self‐paced RT findings

Self‐paced RTs showed no overall difference between conditions across the exploitation segment; instead, consistent with Hypothesis 1, differences emerged at specific frames despite identical visual input. This pattern suggests that spontaneous false‐belief attribution imposes moment‐specific cognitive demands within this viewing context rather than a continuous processing cost. These localized increases in viewing time are consistent with the idea that viewers engage in additional processing when they must reconcile their own privileged knowledge with the character's false belief during interpretation of the unfolding event.

Across clips, the direction of effects was largely consistent (Installation > Control), suggesting greater processing demands when viewers maintain privileged information about a character's false belief. This temporal specificity aligns with event segmentation accounts, which propose that processing costs increase at points where the event model must be updated or enriched (Loschky et al., 2020; Zacks & Swallow, 2007). To situate these effects in time, the frames identified as significant in our analysis were mapped onto specific narrative events within the exploitation phase (see Supporting Information Table S11). Their temporal position within each clip can be inspected in Fig. 3, while Fig. 10 provides a visual depiction of the corresponding moments. Importantly, these interpretations are restricted to frames that survived correction for multiple comparisons, making their convergence with narratively relevant moments unlikely to reflect chance. This is particularly notable given the temporal imprecision inherent to self‐paced viewing: Participants advance through sequences at their own pace, and the stimuli consist of many near‐identical consecutive frames (seven frames per second). As a result, the moment at which belief‐based reasoning occurs can vary across participants, which would be expected to blur effects rather than produce consistent frame‐specific differences.

As an illustrative example, in Clip 4 (“Mistaken Rejection Letter”), the significant frames align with moments at which previously established privileged knowledge becomes relevant for interpreting Harold's actions. The earliest effect (Frame 1) marks the transition into the exploitation phase and initial engagement with the letter outcome, consistent with increased processing at event boundaries where the situation model is established or updated (Gernsbacher, 1990; Loschky et al., 2020; Zacks & Swallow, 2007) and likely reflecting the integration of previously acquired information into the unfolding event (Cohn & Paczynski, 2013). A mid‐phase effect (Frame 70) occurs as Harold handles the letter, where only viewers in the Installation condition can anticipate that he is about to tear up a cheque believing it to be a rejection. A later effect (Frame 87) coincides with him tearing it up without reading it, reinforcing the divergence between viewer knowledge and the character's interpretation.

One exception to the general pattern occurred in Clip 6 (“Mistaken Sheriff Identity”), where the direction of the effect reversed in a specific frame (Frame 184) toward the end of the exploitation phase. In this scene, a man attempts to obtain a permit by persuading Harold—mistaken for the sheriff—to sign it. Earlier in the clip, it is established that the sheriff does not sign such permits. The significant frame occurs when Harold signs the document and the man turns to his partner and winks, indicating that he has successfully obtained the signature. One interpretation is that, in the Control condition, given that viewers treat Harold as the legitimate sheriff, his behavior appears inconsistent with that role: He readily signs the permit, despite the earlier expectation that the sheriff would refuse. Resolving that inconsistency likely increases processing demands. In the Installation condition, by contrast, viewers already know that Harold is not the sheriff, making his behavior expected and reducing additional processing at that moment.

#### 3.3.3 Eye‐Tracking findings across experiments

Across both experiments, the Installation condition was consistently associated with longer fixation durations, indicating increased processing demands and supporting Hypotheses 2a and 3a. In Experiment 1, this effect was limited to fixation duration, with no changes in exploratory metrics. This pattern is consistent with evidence that cognitive load selectively increases fixation duration (Liu et al., 2022) and with the constraints of the self‐paced paradigm, in which each frame typically elicits a single fixation, leaving little opportunity for additional visual exploration. In Experiment 2, during continuous video viewing, longer fixation durations were accompanied by reduced fixation frequency and saccade amplitude, indicating less exploratory and more sustained local processing. Together, these findings are consistent with event‐comprehension accounts in which evolving event models guide information search and constrain attention toward narratively relevant elements (Loschky et al., 2020; Magliano & Zacks, 2011).

Experiment 2 also revealed greater distance from screen center in the Installation condition, suggesting a modest redistribution of attention toward peripheral narratively relevant information. Although distance from screen center provides only a coarse index of spatial allocation, this interpretation is consistent with the exploratory heat map visualizations from Experiment 1, which similarly suggest increased attention to narratively relevant elements.

#### 3.3.4 Exploratory gaze allocation

The exploratory visualizations complement the quantitative eye‐tracking findings by suggesting that spontaneous false‐belief attribution influences not only processing effort but also the allocation of visual attention. In both experiments, viewers with privileged knowledge (Installation condition) appeared to devote greater attention to narratively critical objects than viewers in the Control condition. In Experiment 1, the static heat maps revealed increased attention to narratively critical objects at diagnostic frames. In Clip 4 (“Mistaken Rejection Letter”), this was reflected in increased attention to the letter at the expense of Harold's face, indicating a shift away from the default tendency to prioritize faces during film viewing (Smith, 2006) toward an object critical for interpreting the character's mistaken belief. This attentional pattern converges with the self‐paced reaction‐time effects and with prior recall findings (Cabañas et al., 2023), showing that viewers preferentially represent information known to themselves but not to the character. Experiment 2 extended this observation to continuous viewing: Although the dynamic heat maps summarized gaze over 1‐s windows rather than individual frames, they showed qualitatively similar shifts in attention around the corresponding narrative events despite the strong bottom‐up influence of motion and editing on gaze control (i.e., the “Tyranny of Film”; Loschky et al., 2015). However, these differences were observed only during brief narrative events and are based on qualitative interpretation of heat maps rather than formal statistical comparisons. Accordingly, they should be interpreted as illustrative and require more systematic investigation in future research.

#### 3.3.5 General discussion

The present findings extend event cognition accounts by specifying the role of belief‐based information in shaping ongoing scene comprehension. From this perspective, the Installation condition induces a qualitatively different event model by embedding a belief asymmetry between viewer and character. This asymmetry introduces conflicting belief representations, requiring viewers to maintain and apply a false‐belief representation during unfolding events. The observed increases in fixation duration and viewing time indicate that belief‐based information contributes to processing at moments where it becomes relevant for interpreting character behavior. This pattern aligns with event segmentation accounts in which processing demands increase when event models are updated or enriched (Magliano & Zacks, 2011; Zacks & Swallow, 2007). Taken together, the results suggest that spontaneous belief attribution functions as a component of event model construction, shaping how viewers interpret and anticipate unfolding events under conditions of belief asymmetry.

Importantly, converging evidence from Experiment 2 suggests that several of these effects were also observed during continuous video viewing, despite prior evidence that motion and editing often limit the expression of comprehension‐driven differences in gaze (Hutson et al., 2017; Loschky et al., 2015). This suggests that spontaneous false‐belief attribution may influence visual processing when the cinematic style affords the expression of top‐down influences (e.g., long takes, long shots, and limited camera movement; Loschky et al., 2020).

At the same time, the findings inform ongoing debates about the nature of SToM. The observed increases in processing effort during false‐belief scenarios suggest that maintaining and applying belief representations in dynamic contexts imposes measurable cognitive demands. This pattern is consistent with accounts proposing that belief reasoning recruits additional resources when contextual information must be integrated to interpret others' behavior (Apperly, 2010), as in DI, where viewers must reconcile their own knowledge with the character's false belief. Importantly, evidence for these processing costs has largely been derived from explicit, task‐driven paradigms (e.g., Apperly et al., 2006; Samson et al., 2010). The present findings extend this pattern to spontaneous, unprompted narrative comprehension, showing that comparable processing costs emerge during naturalistic film viewing. This is particularly relevant in light of accounts emphasizing spontaneous cognition as central to real‐world social processing (Kucyi, Tambini, Sadaghiani, Keilholz, & Cohen, 2018), and recent work arguing that instruction‐driven ToM paradigms provide only a partial account of how people spontaneously represent others' mental states during continuous event comprehension (Cabañas 2024).

The paradigm also affords examination of variability in spontaneous mentalizing across individuals, with implications for characterizing individual differences in real‐time social‐cognitive processing. This includes how readily viewers generate and maintain belief representations during DI scenarios in film and how this variability is reflected in attentional dynamics such as fixation duration and saccade patterns. This approach may be particularly informative for studying populations with known differences in social cognition, such as autism, frontotemporal dementia, and other neurodevelopmentally or neurologically diverse populations.

However, several limitations should be noted. While fixation duration indexes processing effort and related eye‐movement measures capture gaze dynamics, these measures do not specify the representational content or function of processing, underscoring the value of combining eye‐tracking with complementary measures of comprehension and recall. A next step would be to integrate content‐based analyses of recall (e.g., mental state references) with eye‐movement data to examine how event model representations relate to attentional allocation. Second, the sample size in Experiment 1 limited statistical power and the scope of analyses, particularly for exploratory visualizations, individual differences, and the detection of effects across multiple dependent measures. Although the sample size was determined based on an a priori power analysis using a large effect size from prior work with the same paradigm (Cabañas et al., 2023), this provides less assurance for the additional measures examined here, for which directly comparable effect size estimates were not available due to differences in task structure and analytic approach. As a result, the study may be underpowered to detect smaller effects in these measures. Future work should examine these effects in larger samples, including designs that allow a more fine‐grained assessment of the temporal dynamics identified here. Experiment 2 addressed this limitation by employing a substantially larger sample, increasing statistical power for detecting effects across eye‐movement measures.

An additional limitation relates to the scope of the stimuli. The use of silent film clips and a small, curated set of scenes may limit generalizability to broader narrative contexts, as the absence of dialogue and reliance on gesture‐based cues may shape attentional patterns and mental state attribution. Extending this approach to audiovisual narratives that include dialogue and sound would allow examination of belief integration under more ecologically representative viewing conditions. A related issue is how these findings map onto real‐life social cognition, where belief asymmetries are a pervasive feature of social interaction, and individuals must continuously track what others know and believe in order to interpret behavior. In this sense, DI provides a highly structured instantiation of this general condition, enabling precise comparison across belief states while holding the underlying events constant. This makes it a tractable model for isolating how spontaneous mentalizing shapes real‐time processing as a first step toward understanding the cognitive architecture of SToM. Future work should examine how these processes operate in naturalistic settings, where belief tracking unfolds in dynamic, interactive environments and is tightly coupled with action and response.

In conclusion, our findings demonstrate that viewers spontaneously construct and maintain complex SToM models when engaging with DI in film. These mental models shape cognitive and attentional processing in real time, as reflected in increased viewing durations, longer fixation durations, and exploratory evidence for preferential attention to narratively relevant objects. These results further indicate that belief‐based information contributes to event model construction and updating, positioning SToM as a component of scene comprehension in dynamic contexts. More broadly, this work contributes to a growing body of research on naturalistic social understanding and highlights the value of DI as a framework for investigating spontaneous mentalizing under conditions that combine experimental control with ecological structure.

## Funding information

This research was supported by a United Kingdom Research and Innovation–Economic and Social Research Council (UKRI‐ESRC) PhD studentship awarded to CC (Grant Reference: ES/P000592/1) and by the Nuffield Foundation (Grant Reference: FR‐000022056). The funders had no role in the design of the study, data collection, analysis, interpretation, or writing of the manuscript.

## Consent to participate

Informed consent was obtained from all individual participants included in the study.

## Consent for publication

Consent for publication of findings and associated data was obtained from all participants included in the study.

## Conflicts of Interest

The authors declare no conflicts of interest.

## Supporting information




**Supporting Information**: cogs70273‐supp‐0001‐SuppMat.docx

## Data Availability

The datasets generated and analyzed during the current study are available from the corresponding author on reasonable request. Scripts used in this study are available upon reasonable request to the corresponding author.

## References

[cogs70273-bib-0075] Achim, A. M. , Guitton, M. , Jackson, P. L. , Boutin, A. , & Monetta, L. (2013). On what ground do we mentalize? Characteristics of current tasks and sources of information that contribute to mentalizing judgments. Psychological Assessment, 25, (1), 117–126. 10.1037/a0029137 22731676

[cogs70273-bib-0001] Apperly, I. (2010). Mindreaders: The cognitive basis of ‘theory of mind’. Hove: Psychology Press.

[cogs70273-bib-0003] Apperly, I. A. , Riggs, K. J. , Simpson, A. , Chiavarino, C. , & Samson, D. (2006). Is belief reasoning automatic? Psychological Science, 17(10), 841–844. 10.1111/j.1467-9280.2006.01791.x 17100782

[cogs70273-bib-0076] Apperly, I. A. (2012). What is “theory of mind”? Concepts, cognitive processes and individual differences. The Quarterly Journal of Experimental Psychology, 65, (5), 825–839. 10.1080/17470218.2012.676055 22533318

[cogs70273-bib-0004] Baayen, R. H. , Davidson, D. J. , & Bates, D. M. (2008). Mixed‐effects modeling with crossed random effects for subjects and items. Journal of Memory and Language, 59(4), 390–412. 10.1016/j.jml.2007.12.005

[cogs70273-bib-0005] Baron‐Cohen, S. , Leslie, A. M. , & Frith, U. (1985). Does the autistic child have a “theory of mind”? Cognition, 21(1), 37–46. 10.1016/0010-0277(85)90022-8 2934210

[cogs70273-bib-0006] Baron‐Cohen, S. , Wheelwright, S. , Hill, J. , Raste, Y. , & Plumb, I. (2001). The “Reading the Mind in the Eyes” Test revised version: A study with normal adults and adults with Asperger syndrome or high‐functioning autism. The Journal of Child Psychology and Psychiatry and Allied Disciplines, 42(2), 241–251. 11280420

[cogs70273-bib-0007] Barr, D. J. , Levy, R. , Scheepers, C. , & Tily, H. J. (2013). Random effects structure for confirmatory hypothesis testing: Keep it maximal. Journal of Memory and Language, 68(3), 255–278. 10.1016/j.jml.2012.11.001 PMC3881361 24403724

[cogs70273-bib-0009] Black, J. E. , & Barnes, J. L. (2015). Fiction and social cognition: The effect of viewing award‐winning television dramas on theory of mind. Psychology of Aesthetics, Creativity and the Arts, 9(4), 423–429. 10.1037/aca0000031

[cogs70273-bib-0010] Cabañas, C. , Senju, A. , & Smith, T. J. (2022). Dramatic irony: A case study in the mutual benefit of combining social neuroscience with film theory. Projections, 16(1), 84–104. 10.3167/proj.2022.160106

[cogs70273-bib-0011] Cabañas, C. , Senju, A. , & Smith, T. J. (2023). The audience who knew too much: Investigating the role of spontaneous theory of mind on the processing of dramatic irony scenes in film. Frontiers in Psychology, 14, 1183660. 10.3389/fpsyg.2023.1183660 PMC10353302 37469900

[cogs70273-bib-0012] Cabañas, C. C. (2024). *Investigating the role of spontaneous theory of mind on the processing of dramatic irony in filmed narratives* (Doctoral dissertation, Birkbeck, University of London). BIROn–Birkbeck Institutional Research Online. 10.18743/PUB.00054770

[cogs70273-bib-0014] Cane, J. E. , Ferguson, H. J. , & Apperly, I. A. (2017). Using perspective to resolve reference: The impact of cognitive load and motivation. Journal of Experimental Psychology: Learning, Memory and Cognition, 43(4), 591–610. 10.1037/xlm0000345 28068126

[cogs70273-bib-0015] Castano, E. (2021). Art films foster theory of mind. Humanities and Social Sciences Communications, 8, 119. 10.1057/s41599-021-00793-y

[cogs70273-bib-0016] Castelhano, M. S. , Mack, M. L. , & Henderson, J. M. (2009). Viewing task influences eye movement control during active scene perception. Journal of Vision, 9(3), 6–6. 10.1167/9.3.6 19757945

[cogs70273-bib-0017] Chung, Y. S. , Barch, D. , & Strube, M. (2014). A meta‐analysis of mentalizing impairments in adults with schizophrenia and autism spectrum disorder. Schizophrenia Bulletin, 40(3), 602–616. 10.1093/schbul/sbt048 PMC3984506 23686020

[cogs70273-bib-0018] Cohn, N. (2016). From visual narrative grammar to filmic narrative grammar: The narrative structure of static and moving images. In J. Wildfeuer & J. A. Bateman (Eds.), Film text analysis (pp. 94–117). New York: Routledge.

[cogs70273-bib-0019] Cohn, N. , & Paczynski, M. (2013). Prediction, events and the advantage of Agents: The processing of semantic roles in visual narrative. Cognitive Psychology, 67(3), 73–97. 10.1016/j.cogpsych.2013.07.002 PMC3895484 23959023

[cogs70273-bib-0021] Csibra, G. , & Gergely, G. (1998). The teleological origins of mentalistic action explanations: A developmental hypothesis. Developmental Science, 1(2), 255–259. 10.1111/1467-7687.00039

[cogs70273-bib-0022] Csibra, G. , & Gergely, G. (2007). ‘Obsessed with goals’: Functions and mechanisms of teleological interpretation of actions in humans. Acta Psychologica, 124(1), 60–78. 10.1016/j.actpsy.2006.09.007 17081489

[cogs70273-bib-0082] Faul, F. , Erdfelder, E. , Buchner, A. , & Lang, A.‐G. (2009). Statistical power analyses using G*Power 3.1: Tests for correlation and regression analyses. Behavior Research Methods, 41, (4), 1149–1160. 10.3758/BRM.41.4.1149 19897823

[cogs70273-bib-0084] Gernsbacher, M. A. (1990). Language comprehension as structure building. Lawrence Erlbaum Associates.

[cogs70273-bib-0025] Haensel, J. X. , Danvers, M. , Ishikawa, M. , Itakura, S. , Tucciarelli, R. , Smith, T. J. , & Senju, A. (2020). Culture modulates face scanning during dyadic social interactions. Scientific Reports, 10(1), 1958. 10.1038/s41598-020-58802-0 PMC7005015 32029826

[cogs70273-bib-0026] Happé, F. G. E. (1994). An advanced test of theory of mind: Understanding of story characters’ thoughts and feelings by able autistic, mentally handicapped and normal children and adults. Journal of Autism and Developmental Disorders, 24(2), 129–154. 10.1007/BF02172093 8040158

[cogs70273-bib-0081] Hasson, U. , Nir, Y. , Levy, I. , Fuhrmann, G. , & Malach, R. (2004). Intersubject synchronization of cortical activity during natural vision. Science, 303, (5664), 1634–1640. 10.1126/science.1089506 15016991

[cogs70273-bib-0079] Henderson, J. M. (2003). Human gaze control during real‐world scene perception. Trends in Cognitive Sciences, 7, (11), 498–504. 10.1016/j.tics.2003.09.006 14585447

[cogs70273-bib-0028] Huettig, F. , Rommers, J. , & Meyer, A. S. (2011). Using the visual world paradigm to study language processing: A review and critical evaluation. Acta Psychologica, 137(2), 151–171. 10.1016/j.actpsy.2010.11.003 21288498

[cogs70273-bib-0029] Huff, M. , Maurer, A. E. , Brich, I. R. , Pagenkopf, A. , Wickelmaier, F. , & Papenmeier, F. (2018). Construction and updating of event models in auditory event processing. Journal of Experimental Psychology: Learning, Memory and Cognition, 44(2), 307–320. 10.1037/xlm0000482 28933900

[cogs70273-bib-0030] Hutson, J. P. , Chandran, P. , Magliano, J. P. , Smith, T. J. , & Loschky, L. C. (2022). Narrative comprehension guides eye movements in the absence of motion. Cognitive Science, 46(5), e13131. 10.1111/cogs.13131 35579883

[cogs70273-bib-0031] Hutson, J. P. , Smith, T. J. , Magliano, J. P. , & Loschky, L. C. (2017). What is the role of the film viewer? The effects of narrative comprehension and viewing task on gaze control in film. Cognitive Research: Principles and Implications, 2(1), 46. 10.1186/s41235-017-0080-5 PMC5698392 29214207

[cogs70273-bib-0032] Keysar, B. , Lin, S. , & Barr, D. J. (2003). Limits on theory of mind use in adults. Cognition, 89(1), 25–41. 10.1016/s0010-0277(03)00064-7 12893123

[cogs70273-bib-0033] Kingstone, A. , Smilek, D. , & Eastwood, J. D. (2008). Cognitive ethology: A new approach for studying human cognition. British Journal of Psychology, (London, England: 1953), 99(Pt 3), 317–340. 10.1348/000712607x251243 17977481

[cogs70273-bib-0034] Kopatich, R. D. , Feller, D. P. , Kurby, C. A. , & Magliano, J. P. (2019). The role of character goals and changes in body position in the processing of events in visual narratives. Cognitive Research: Principles and Implications, 4(1), 22. 10.1186/s41235-019-0176-1 PMC6614232 31286278

[cogs70273-bib-0035] Kovács, Á. M. , Téglás, E. , & Endress, A. D. (2010). The social sense: Susceptibility to others’ beliefs in human infants and adults. Science, (New York, N.Y.), 330(6012), 1830–1834. 10.1126/science.1190792 21205671

[cogs70273-bib-0036] Kucyi, A. , Tambini, A. , Sadaghiani, S. , Keilholz, S. , & Cohen, J. R. (2018). Spontaneous cognitive processes and the behavioral validation of time‐varying brain connectivity. Network Neuroscience, 2(4), 397–417. 10.1162/netn_a_00037 PMC6195165 30465033

[cogs70273-bib-0037] Lavandier, Y. (2005). Writing drama: A comprehensive guide for playwrights and scriptwriters. Cergy Cedex, France: Le Clown & l'Enfant.

[cogs70273-bib-0038] Levin, D. T. , Hymel, A. M. , & Baker, L. (2013). Belief, desire, action and other stuff: Theory of mind in movies. In A. P. Shimamura (Ed.), Psychocinematics: Exploring cognition at the movies (pp. 244–266). Oxford, England: Oxford University Press. 10.1093/acprof:oSo/9780199862139.003.0013

[cogs70273-bib-0039] Levin, D. T. , Mattarella‐Micke, A. , Lee, M. J. , Baker, L. J. , Bezdek, M. A. , & McCandliss, B. D. (2022). How movie events engage children's brains to combine visual attention with domain‐specific processing involving number and theory of mind in a cinematic arena. Projections, 16(1), 67–83. 10.3167/proj.2022.160105

[cogs70273-bib-0040] Liu, J.‐C. , Li, K.‐A. , Yeh, S.‐L. , & Chien, S.‐Y. (2022). Assessing perceptual load and cognitive load by fixation‐related information of eye movements. Sensors, 22(3), 1187. 10.3390/s22031187 PMC8839381 35161930

[cogs70273-bib-0041] Loschky, L. C. , Larson, A. M. , Magliano, J. P. , & Smith, T. J. (2015). What would Jaws do? The Tyranny of film and the relationship between gaze and higher‐level narrative film comprehension. PLOS ONE, 10(11), e0142474. 10.1371/journal.pone.0142474 PMC4659561 26606606

[cogs70273-bib-0042] Loschky, L. C. , Larson, A. M. , Smith, T. J. , & Magliano, J. P. (2020). The scene perception & event comprehension theory (SPECT) applied to visual narratives. Topics in Cognitive Science, 12(1), 311–351. 10.1111/tops.12455 PMC9328418 31486277

[cogs70273-bib-0043] Luke, S. G. , & Henderson, J. M. (2016). The influence of content meaningfulness on eye movements across tasks: Evidence from scene viewing and reading. Frontiers in Psychology, 7, 257. 10.3389/fpsyg.2016.00257 PMC4771774 26973561

[cogs70273-bib-0044] Magliano, J. P. , Miller, J. , & Zwaan, R. A. (2001). Indexing space and time in film understanding. Applied Cognitive Psychology, 15(5), 533–545. 10.1002/acp.724

[cogs70273-bib-0045] Magliano, J. P. , Yan, E. F. , Ackerman, T. , Mccarthy, K. S. , & Kurby, C. A. (2024). Understanding the role of cinematic features on the experience of filmed events. Projections, 18(3), 13–40. 10.3167/proj.2024.180302

[cogs70273-bib-0046] Magliano, J. P. , & Zacks, J. M. (2011). The Impact of continuity editing in narrative film on event segmentation. Cognitive Science, 35(8), 1489–1517. 10.1111/j.1551-6709.2011.01202.x PMC3208769 21972849

[cogs70273-bib-0047] Matuschek, H. , Kliegl, R. , Vasishth, S. , Baayen, R. H. , & Bates, D. (2017). Balancing Type I error and power in linear mixed models. Journal of Memory and Language, 94, 305–315. 10.1016/j.jml.2017.01.001

[cogs70273-bib-0048] Meins, E. , Fernyhough, C. , & Harris‐Waller, J. (2014). Is mind‐mindedness trait‐like or a quality of close relationships? Evidence from descriptions of significant others, famous people and works of art. Cognition, 130(3), 417–427. 10.1016/j.cognition.2013.11.009 24448488

[cogs70273-bib-0078] Newmeyer, F. C. , & Taylor, S. (1924). Girl Shy [Film]. Harold Lloyd Corporation.

[cogs70273-bib-0050] Nuthmann, A. , Smith, T. J. , Engbert, R. , & Henderson, J. M. (2010). CRISP: A computational model of fixation durations in scene viewing. Psychological Review, 117(2), 382–405. 10.1037/a0018924 20438231

[cogs70273-bib-0051] O'Neill, D. K. , & Shultis, R. M. (2007). The emergence of the ability to track a character's mental perspective in narrative. Developmental Psychology, 43(4), 1032–1037. 10.1037/0012-1649.43.4.1032 17605533

[cogs70273-bib-0052] Papenmeier, F. , Boss, A. , & Mahlke, A.‐K. (2019). Action goal changes caused by agents and patients both induce global updating of event models. Journal of Experimental Psychology: Learning, Memory and Cognition, 45(8), 1441–1454. 10.1037/xlm0000651 30070563

[cogs70273-bib-0054] Pedziwiatr, M. A. , Heer, S. , Coutrot, A. , Bex, P. , & Mareschal, I. (2023). Prior knowledge about events depicted in scenes decreases oculomotor exploration. Cognition, 238, 105544. 10.1016/j.cognition.2023.105544 PMC13035412 37419068

[cogs70273-bib-0055] Perner, J. (1991). Understanding the representational mind. Cambridge: The MIT Press.

[cogs70273-bib-0056] Persson, P. (2003). Understanding cinema: A psychological theory of moving imagery. Cambridge: Cambridge University Press.

[cogs70273-bib-0057] Quesque, F. , Apperly, I. , Baillargeon, R. , Baron‐Cohen, S. , Becchio, C. , Bekkering, H. , Bernstein, D. , Bertoux, M. , Bird, G. , Bukowski, H. , Burgmer, P. , Carruthers, P. , Catmur, C. , Dziobek, I. , Epley, N. , Erle, T. M. , Frith, C. , Frith, U. , Galang, C. M. , Gallese, V. , Grynberg, D. , Happé, F. , Hirai, M. , Hodges, S. D. , Kanske, P. , Kret, M. , Lamm, C. , Nandrino, J. L. , Obhi, S. , Olderbak, S. , Perner, J. , Rossetti, Y. , Schneider, D. , Schurz, M. , Schuwerk, T. , Sebanz, N. , Shamay‐Tsoory, S. , Silani, G. , Spaulding, S. , Todd, A. R. , Westra, E. , Zahavi, D. , & Brass, M. (2024). Defining key concepts for mental state attribution. Communications Psychology, 2(1), 29. 10.1038/s44271-024-00077-6 PMC11332223 39242813

[cogs70273-bib-0059] Rapp, D. N. , & Gerrig, R. J. (2006). Predilections for narrative outcomes: The impact of story contexts and reader preferences. Journal of Memory and Language, 54(1), 54–67. 10.1016/j.jml.2005.04.003

[cogs70273-bib-0060] Raz, G. , Jacob, Y. , Gonen, T. , Winetraub, Y. , Flash, T. , Soreq, E. , & Hendler, T. (2014). Cry for her or cry with her: Context‐dependent dissociation of two modes of cinematic empathy reflected in network cohesion dynamics. Social Cognitive and Affective Neuroscience, 9(1), 30–38. 10.1093/scan/nst052 PMC3871736 23615766

[cogs70273-bib-0061] Richardson, H. , Lisandrelli, G. , Riobueno‐Naylor, A. , & Saxe, R. (2018). Development of the social brain from age three to twelve years. Nature Communications, 9, 1027. 10.1038/s41467-018-03399-2 PMC5847587 29531321

[cogs70273-bib-0062] Rooney, B. , & Bálint, K. E. (2018). Watching more closely: Shot scale affects film viewers’ theory of mind tendency but not ability. Frontiers in Psychology, 8, 2349. https://www.frontiersin.org/articles/10.3389/fpsyg.2017.02349 10.3389/fpsyg.2017.02349 PMC5776141 29387032

[cogs70273-bib-0063] Samson, D. , Apperly, I. A. , Braithwaite, J. J. , Andrews, B. J. , & Bodley Scott, S. E. (2010). Seeing it their way: Evidence for rapid and involuntary computation of what other people see. Journal of Experimental Psychology. Human Perception and Performance, 36(5), 1255–1266. 10.1037/a0018729 20731512

[cogs70273-bib-0064] Senju, A. (2012). Spontaneous theory of mind and its absence in autism spectrum disorders. The Neuroscientist, 18(2), 108–113. 10.1177/1073858410397208 PMC3796729 21609942

[cogs70273-bib-0065] Smith, T. J. (2006). An attentional theory of continuity editing (Doctoral dissertation, University of Edinburgh). https://era.ed.ac.uk/handle/1842/1076

[cogs70273-bib-0066] Smith, T. J. (2012). The attentional theory of cinematic continuity. Projections, 6(1), 1–27.

[cogs70273-bib-0067] Smith, T. J. , & Mital, P. K. (2013). Attentional synchrony and the influence of viewing task on gaze behavior in static and dynamic scenes. *Journal of Vision*, 13(8), 16, 1–24. 10.1167/13.8.16. 23863509

[cogs70273-bib-0069] Symeonidou, I. , Dumontheil, I. , Chow, W.‐Y. , & Breheny, R. (2016). Development of online use of theory of mind during adolescence: An eye‐tracking study. Journal of Experimental Child Psychology, 149, 81–97. 10.1016/j.jecp.2015.11.007 26723471

[cogs70273-bib-0080] Tseng, C.‐I. , Laubrock, J. , & Bateman, J. A. (2021). The impact of multimodal cohesion on attention and interpretation in film. Discourse, Context & Media, 44, 100544. 10.1016/j.dcm.2021.100544

[cogs70273-bib-0077] Vanderwal, T. , Eilbott, J. , & Castellanos, F. X. (2019). Movies in the magnet: Naturalistic paradigms in developmental functional neuroimaging. Developmental Cognitive Neuroscience, 36, 100600. 10.1016/j.dcn.2018.10.004 PMC6969259 30551970

[cogs70273-bib-0070] Võ, M. L.‐H. , Smith, T. J. , Mital, P. K. , & Henderson, J. M. (2012). Do the eyes really have it? Dynamic allocation of attention when viewing moving faces. Journal of Vision, 12(13), 3. 10.1167/12.13.3 23211270

[cogs70273-bib-0083] Westfall, J. , Kenny, D. A. , & Judd, C. M. (2014). Statistical power and optimal design in experiments in which samples of participants respond to samples of stimuli. Journal of Experimental Psychology: General, 143, (5), 2020–2045. 10.1037/xge0000014 25111580

[cogs70273-bib-0072] Whitney, C. , Huber, W. , Klann, J. , Weis, S. , Krach, S. , & Kircher, T. (2009). Neural correlates of narrative shifts during auditory story comprehension. NeuroImage, 47(1), 360–366. 10.1016/j.neuroimage.2009.04.037 19376237

[cogs70273-bib-0073] Wimmer, H. , & Perner, J. (1983). Beliefs about beliefs: Representation and constraining function of wrong beliefs in young children's understanding of deception. Cognition, 13(1), 103–128. 10.1016/0010-0277(83)90004-5 6681741

[cogs70273-bib-0074] Zacks, J. M. , & Swallow, K. M. (2007). Event segmentation. Current Directions in Psychological Science, 16(2), 80–84. 10.1111/j.1467-8721.2007.00480.x PMC3314399 22468032

