# Supplementary material for: Viewers Actively Extract and Maintain Spontaneous Theory of Mind Representations in Dramatic Irony Scenes
Source: Cogn Sci. 2026 Oct 30;50(11):e70273. doi: 10.1111/cogs.70273 (PMC13721779; doi:10.1111/cogs.70273)
Supplement: Supplementary file 1 [file COGS-50-e70273-s001.docx]

**Supplementary Materials**

## **Supplementary Text 1**

***Deviations from Pre-Registered Analysis***

The analysis plan for the self-paced RTs was prespecified in a preregistration document that can be found on the Open Science Framework [https://osf.io/by56s]. In order to investigate how deeper cognitive processing is reflected in participants' gaze and pupil size, we also collected eye-tracking and pupillometry data. We pre-specified that the results from the Self-Paced Viewing paradigm's reaction time analysis would inform the secondary analysis of eye-tracking data. However, due to the complexities of individual and frame-specific varying reaction times and slow changes in pupil size, pupillometry data was not analysed in this study. Moreover, motion stimuli (film viewing) can induce pupillary constriction as a confounding variable, making it advisable to use shorter length clips than those available in our film clip corpus (Mathôt & Vilotijević, 2022). Moreover, controlling for these factors becomes more challenging in the Self-Paced Viewing paradigm, where the length of each trial is variable.

## **Supplementary Text 2**

## ***Description of Clips***

***Clip 1 – ‘Fake Injury Scheme’ from Never Weaken Part 1 (1921)***

***Establisher*** (0:00–1:18) : Harold wants to help his friend or love interest, who will get fired from the osteopathic clinic where she works if they do not get more clients.

***Installation*** (1:18–2:08): Harold runs into an acrobat and they come up a plan together.

***Exploitation* *(Control: 1:18–2:21; Installation: 2:08–2:21):*** An acrobat appears to fall and be injured in front of a crowd and Harold presents himself as someone who can treat him. The crowd gathers and shows interest in the clinic.

***Victim of dramatic irony***: the people in the crowd whose goal is to get a treatment for their illnesses or disabilities.

***Clip 2 – ‘False Team Membership’ from The Freshman (1925)***

***Establisher*** (0:00–1:32): Harold out for the team to be a football player

***Installation*** (1:32–2:01): After tryouts, Harold is unaware that he did not make the team. The coach and teammate trick him into thinking he made the team but assign him the role of water boy.

**Exploitation (Control: 1:32–3:26; Installation: 2:01–3:26):** Harold tells a girl he made the team and goes to the field expecting to play.

***Victim of dramatic irony***: Harold whose goal is to be a football player.

***Clip 3 – ‘Jealous Misunderstanding’ from Never Weaken (1921)***

***Establisher*** (0:00–0:44): Harold proposes to a girl and she accepts.

***Installation*** (0:44–1:01): In another scene, the girl is talking to a man who is actually her brother and he's an ordained minister.

***Exploitation (Control: 0:44–1:25; Installation: 1:01–1:25):*** Harold observes an interaction between the girl and the man and reacts emotionally before leaving.

***Victim of dramatic irony***: Harold whose goal is to marry the girl.

***Clip 4 – ‘Mistaken Rejection Letter’ from Girl Shy (1924)***

***Establisher*** (0:00–1:44): Harold visits a publishing house to inquire about the possibility of publishing his book. However, the publisher finds his book to be extremely comical, so they reject it and inform Harold that he will receive a rejection letter in the mail.

***Installation*** (1:44–2:23): When Harold leaves, a senior employee convinces the editor to reconsider and publish the manuscript as a comedy. He then instructs the employee to send a check to Harold instead of the rejection letter.

***Exploitation (Control: 1:44–3:06; Installation: 2:23–3:06):*** Harold receives a letter, appears disappointed and tears it apart without opening it.

***Victim of dramatic irony***: Harold whose goal is to publish his book and make money with it.

***Clip 5 –‘Accidental Donation’ from For Heaven’s Sake (1926)***

***Establisher*** (0:00–2:48): A missionary and his daughter need to raise money for a homeless mission and decide to write a letter to Harold, who is portrayed as a wealthy man.

***Installation*** (2:48–3:51): Harold accidentally burns down a cart that belongs to the missionary.

***Exploitation (Control: 2:48–4:12; Installation: 3:51–4:12):*** Harold gives the missionary money and the missionary and his daughter respond with gratitude.

***Victim of dramatic irony***: the missionary and his daughter, whose goal is to raise money for the mission.

***Clip 6 – ‘Mistaken Sheriff Identity’ from The Kid Brother (1927)***

***Establisher*** (0:00–0:26): Two men running a traveling show are seeking a permit from the sheriff.

***Installation*** (0:26–4:11): Harold feels inferior to his brothers and is not allowed to go to town with them. He stays at home and dresses up as his father, who is the sheriff.

***Exploitation (Control: 0:26–5:18; Installation: 4:11–5:18):*** The two men encounter Harold dressed as the sheriff and obtain a signed permit from him.

***Victim of dramatic irony***: The two men, whose goal is to get the permit for their show.

**Supplementary Figure S1.** *Example stills from Clip 1 (“Fake Injury Scheme”).*

*Note.* Stills taken with permission from Never Weaken (Copyright of the Harold Lloyd Trust, 1921).

**Supplementary Figure S2.** *Example stills from Clip 2 (“False Team Membership”).*

*Note.* Stills taken with permission from The Freshman (Copyright of the Harold Lloyd Trust, 1925).

**Supplementary Figure S3.** *Example stills from Clip 3 (“Jealous Misunderstanding”).*

*Note.* Stills taken with permission from Never Weaken (Copyright of the Harold Lloyd Trust, 1921).

**Supplementary Figure S4.** *Example stills from Clip 4 (“Mistaken Rejection Letter”).*

*Note.* Stills taken with permission from Girl Shy (Copyright of the Harold Lloyd Trust, 1924).

**Supplementary Figure S5.** *Example stills from Clip 5 (“Accidental Donation”).*

*Note.* Stills taken with permission from For Heaven’s Sake (Copyright of the Harold Lloyd Trust, 1926).

**Supplementary Figure S6.** *Example stills from Clip 6 (“Mistaken Sheriff Identity”).*

*Note.* Stills taken with permission from The Kid Brother (Copyright of the Harold Lloyd Trust, 1927).

## **Supplementary Figure S7**. *Static* *Exploratory Illustration of Gaze Allocation: Clip 1 - "Fake Injury Scheme"*

## *Note.* Narratively Critical Objects/Characters on the right and difference fixation heat maps between Installation and Control groups for Frame 192 of Clip 1 - Fake Injury Scheme. Colours show the fixation duration contrast: warmer colours indicate longer fixations by the Installation group (in comparison to the *control* group); cooler colours show longer fixations by the *control* group (in comparison the *installation* group).

## **Supplementary Figure S8*.*** *Static* *Exploratory Illustration of Gaze Allocation: Clip 2 – "False Team Membership”.*

## *Note.* Narratively Critical Objects/Characters on the right and difference fixation heat maps between Installation and Control groups for Frame 43 of Clip 2 - False Team Membership. Colours show the fixation duration contrast: warmer colours indicate longer fixations by the Installation group (in comparison to the control group); cooler colours show longer fixations by the control group (in comparison the installation group).

## **Supplementary Figure S9.** *Static* *Exploratory Illustration of Gaze Allocation: Clip 6 - "Mistaken Sheriff Identity"*

## *Note.* Narratively Critical Objects/Characters on the right and difference fixation heat maps between Installation and Control groups for Frame 184 of Clip 6. Colours show the fixation duration contrast: warmer colours indicate longer fixations by the Installation group (in comparison to the control group); cooler colours show longer fixations by the control group (in comparison the installation group).

**Supplementary Figure S10.** *Dynamic Exploratory Illustration of Gaze Allocation to Narratively Critical Elements: Clip 1 – "Acrobat"*

*Note.* Left: fixation heat map for the Control condition. Right: fixation heat map for the Installation condition. Bottom: difference heat map (Installation − Control) for the one-second window centred on the visual event corresponding to Frame 192 identified in Experiment 1 (±12 video frames). Warmer colours indicate relatively longer fixation durations in the Installation condition, whereas cooler colours indicate relatively longer fixation durations in the Control condition.

**Supplementary Figure S11.** *Dynamic Exploratory Illustration of Gaze Allocation to Narratively Critical Elements: Clip 2 – "Football"*

***Note.* Left:** fixation heat map for the **Control** condition**. Right:** fixation heat map for the **Installation** condition. **Bottom:** difference heat map (Installation − Control) for the one-second window centred on the visual event corresponding to **Frame 43** identified in Experiment 1 (±12 video frames). Warmer colours indicate relatively longer fixation durations in the Installation condition, whereas cooler colours indicate relatively longer fixation durations in the Control condition.

**Supplementary Figure S12.** *Dynamic* *Exploratory Illustration of Gaze Allocation to Narratively Critical Elements: Clip 4 – "Mistaken Rejection Letter" (Early Event)*

*Note.* Left: fixation heat map for the Control condition. Right: fixation heat map for the Installation condition. Bottom: difference heat map (Installation − Control) for the one-second window centred on the visual event corresponding to Frame 70 identified in Experiment 1 (±12 video frames). Warmer colours indicate relatively longer fixation durations in the Installation condition, whereas cooler colours indicate relatively longer fixation durations in the Control condition.

**Supplementary Figure S13.** *Exploratory Illustration of Gaze Allocation to Narratively Critical Elements: Clip 4 – "Mistaken Rejection Letter" (Late Event)*

***Note.*** **Left:** fixation heat map for the **Control** condition**. Right:** fixation heat map for the **Installation** condition. **Bottom:** difference heat map (Installation − Control) for the one-second window centred on the visual event corresponding to **Frame 87** identified in Experiment 1 (±12 video frames). Warmer colours indicate relatively longer fixation durations in the Installation condition, whereas cooler colours indicate relatively longer fixation durations in the Control condition.

**Supplementary Figure S14.** *Exploratory Illustration of Gaze Allocation to Narratively Critical Elements: Clip 6 – "Mistaken Sheriff Identity"*

***Note.* Left:** fixation heat map for the **Control** condition. **Right:** fixation heat map for the **Installation** condition. **Bottom:** difference heat map (Installation − Control) for the one-second window centred on the visual event corresponding to **Frame 184** identified in Experiment 1 (±12 video frames). Warmer colours indicate relatively longer fixation durations in the Installation condition, whereas cooler colours indicate relatively longer fixation durations in the Control condition.

**Supplementary Table S1.** *Mean self-paced reaction times (ms) by clip and condition*

| Clip | Label | Control M (SD) | Installation M (SD) |
| --- | --- | --- | --- |
| 1 | Fake Injury Scheme | **423 (191)** | **432 (216)** |
| 2 | False Football Membership | **375 (173)** | **422 (198)** |
| 3 | Jealous Misunderstanding | **365 (183)** | **352 (185)** |
| 4 | Mistaken Rejection Letter | **362 (153)** | **370 (186)** |
| 5 | Accidental Donation | **419 (177)** | **394 (189)** |
| 6 | Mistaken Sheriff Identity | **371 (167)** | **358 (179)** |

*Note.* Values reflect frame-level self-paced reaction times (ms), averaged within clip and condition after preprocessing.

**Supplementary Table S2.** *Model comparison for linear mixed-effects analyses of self-paced reaction times across all clips*

| Model | Formula | Comparison | χ² | df | AIC | BIC | p-value |
| --- | --- | --- | --- | --- | --- | --- | --- |
| M0 | logRT ~ 1 + (1 \| participant) | baseline | — | — | 18639 | 18663 | — |
| M1 | logRT ~ 1 + (1 \| participant) + (1 \| clip) | + clip intercept | 985.86 | 1 | 17655 | 17687 | < .001 |
| M2 | logRT ~ condition + (1 \| participant) + (1 \| clip) | + condition | 0.73 | 1 | 17656 | 17697 | .393 |
| M3 | logRT ~ condition + frame + (1 \| participant) + (1 \| clip) | + frame | 284.05 | 1 | 17374 | 17423 | < .001 |
| M4 | logRT ~ condition * frame + (1 \| participant) + (1 \| clip) | + condition × frame | 1.70 | 1 | 17374 | 17431 | .192 |

*Note.* The table details the model comparisons, degrees of freedom (df), Akaike Information Criterion (AIC), Bayesian Information Criterion (BIC), log-likelihood (logLik), test comparisons, likelihood ratio (L.Ratio) and p-values.

**Supplementary Table S3.** *Test of clip-specific variation in condition effects on self-paced reaction times*

| Model | | Formula | Comparison | χ² | df | AIC | BIC | p-value |
| --- | --- | --- | --- | --- | --- | --- | --- | --- |
| M2 | logRT ~ condition + (1 \| participant) + (1 \| clip) | | reference | — | — | 17656 | 17697 | — |
| M5 | logRT ~ condition * clip + (1 \| participant) + (1 \| clip) | | + condition × clip | 199.89 | 10 | 17476 | 17598 | < .001 |

**Supplementary Table S4.** *Fixed-effect estimates for the final linear mixed-effects model of self-paced RTs*

| Effect | β | SE | t | p |
| --- | --- | --- | --- | --- |
| Intercept | 5.856 | 0.103 | 57.05 | < .001 |
| Condition (With) | 0.124 | 0.135 | 0.92 | .367 |
| Frame | -0.00046 | 0.00004 | -11.90 | < .001 |
| Condition × Frame | -0.00007 | 0.00005 | -1.30 | .192 |

**Supplementary Table S5.** *Summary of final clip-specific models for self-paced RTs*

| **Clip** | **Final model formula** | **AIC** | **BIC** | **logLik** | **L.Ratio** | **p-value** |
| --- | --- | --- | --- | --- | --- | --- |
| 1 | RT ~ condition * frame + (1 \| participant) | 3531.052 | 6947.176 | -1256.526 | 294.121 | 0.035* |
| 2 | RT ~ condition * frame + (1 \| participant) | 2135.644 | 6586.264 | -426.822 | 402.394 | <.001*** |
| 3 | RT ~ condition * frame + (1 \| participant) | 1030.415 | 1800.985 | -373.2075 | 81.4741 | 0.145 |
| 4 | RT ~ condition * frame + (1 \| participant) | 1590.056 | 2983.992 | -560.0278 | 203.0367 | <.001*** |
| 5 | RT ~ condition * frame + (1 \| participant) | 2056.135 | 3816.559 | -741.0676 | 142.4028 | 0.4511 |
| 6 | RT ~ condition * frame + (1 \| participant) | 2364.051 | 5094.084 | -763.026 | 241.721 | 0.049* |

*Note*. The table presents the final model for each clip, alongside key statistical metrics including Akaike Information Criterion (AIC), Bayesian Information Criterion (BIC), log-likelihood (logLik), likelihood ratio (L.Ratio) and p-values. Significance levels are denoted with asterisks: * p < 0.05, ** p < 0.01, *** p < 0.001.

**Supplementary Table S6.** *Frame-level condition contrasts for Clip 1* - Fake Injury Scheme *(self-paced RTs)*

| **Frame** | **β** | **SE** | **df** | **t-ratio** | **p-value** |
| --- | --- | --- | --- | --- | --- |
| 2 | -0.64357 | 0.213 | 22 | -3.026 | 0.006* |
| 22 | -0.47060 | 0.213 | 22 | -2.213 | 0.038* |
| 55 | -0.61968 | 0.213 | 22 | -2.914 | 0.008* |
| 148 | -0.46862 | 0.213 | 22 | -2.203 | 0.038* |
| 192 | -0.52373 | 0.213 | 22 | -2.463 | 0.022* |

Note. Significance levels are denoted with asterisks: * p < 0.05, ** p < 0.01, *** p < 0.001.

**Supplementary Table S7.** *Frame-level condition contrasts for Clip 2 - False Team Membership (self-paced RTs)*

| **Frame** | **β** | **SE** | **df** | **t-ratio** | **p-value** |
| --- | --- | --- | --- | --- | --- |
| 1 | -0.536746 | 0.201 | 22 | -2.668 | 0.014* |
| 16 | -0.422440 | 0.201 | 22 | -2.100 | 0.047* |
| 19 | -0.439594 | 0.201 | 22 | -2.185 | 0.039* |
| 43 | -0.453654 | 0.201 | 22 | -2.255 | 0.034* |

Note. Significance levels are denoted with asterisks: * p < 0.05, ** p < 0.01, *** p < 0.001.

**Supplementary Table S8.** *Frame-level condition contrasts for Clip 4 - Mistaken Rejection Letter (self-paced RTs)*

| **Frame** | **Β** | **SE** | **df** | **t-ratio** | **p-value** |
| --- | --- | --- | --- | --- | --- |
| 1 | -0.76376 | 0.204 | 22 | -3.739 | 0.001*** |
| 70 | -0.52290 | 0.204 | 22 | -2.565 | 0.018* |
| 87 | -0.48507 | 0.204 | 22 | -2.375 | 0.027* |

Note. Significance levels are denoted with asterisks: * p < 0.05, ** p < 0.01, *** p < 0.001.

**Supplementary Table S9.** *Frame-level condition contrasts for Clip 6 (self-paced RTs)*

| **Frame** | **β** | **SE** | **df** | **t-ratio** | **p-value** |
| --- | --- | --- | --- | --- | --- |
| 1 | -0.489428 | 0.204 | 22 | -2.396 | 0.025* |
| 2 | -0.633976 | 0.204 | 22 | -3.103 | 0.005* |
| 77 | -0.514155 | 0.204 | 22 | -2.517 | 0.019* |
| 184 | 0.495020 | 0.204 | 22 | 2.423 | 0.024* |

Note. Significance levels are denoted with asterisks: * p < 0.05, ** p < 0.01, *** p < 0.001.

**Supplementary Table S10.** *Counterbalancing Scheme for Experiment 2*

**Group 1 (2 Installation / 3 Control)**

| Order | V1 | V2 | V3 | V4 | V5 |
| --- | --- | --- | --- | --- | --- |
| 1 | Installation | Installation | Control | Control | Control |
| 2 | Installation | Installation | Control | Control | Control |
| 3 | Control | Control | Installation | Installation | Control |
| 4 | Control | Control | Installation | Installation | Control |
| 5 | Installation | Control | Installation | Control | Control |
| 6 | Installation | Control | Installation | Control | Control |
| 7 | Control | Installation | Control | Installation | Control |
| 8 | Control | Installation | Control | Installation | Control |

**Group 2 (3 Installation / 2 No Installation)**

| Order | V1 | V2 | V3 | V4 | V5 |
| --- | --- | --- | --- | --- | --- |
| 1 | Control | Control | Installation | Installation | Installation |
| 2 | Control | Control | Installation | Installation | Installation |
| 3 | Installation | Installation | Control | Control | Installation |
| 4 | Installation | Installation | Control | Control | Installation |
| 5 | Control | Installation | Control | Installation | Installation |
| 6 | Control | Installation | Control | Installation | Installation |
| 7 | Installation | Control | Installation | Control | Installation |
| 8 | Installation | Control | Installation | Control | Installation |

*Note.* Group 1 and Group 2 implement complementary list structures: participants viewed either two Installation and three No Installation clips or the inverse. Across participants, each clip appears equally often in both conditions.

**Supplementary Table S11.** *Significant frames mapped onto narrative events during the exploitation phase*

| Clip (Label) | Frame | Direction | Narrative event (interpretation) |
| --- | --- | --- | --- |
| 1 – Fake Injury Scheme | 2 | I > C | Transition into exploitation; acrobat’s staged injury is introduced |
|  | 22 | I > C | Crowd gathers around the injured acrobat |
|  | 55 | I > C | Harold is treated by the crowd as an osteopath |
|  | 148 | I > C | The acrobat’s rapid recovery reinforces the apparent success of Harold’s treatment |
|  | 192 | I > C | A crowd member approaches Harold to request business cards |
| 2 – False Team Membership | 1 | I > C | Transition into exploitation; Harold climbs the stairs, visibly exhausted |
|  | 16 | I > C | Harold’s girlfriend approaches him as he climbs the stairs |
|  | 19 | I > C | Harold turns to speak to the girl |
|  | 43 | I > C | Harold tells the girl he made the team excitedly |
| 4 – Mistaken Rejection Letter | 1 | I > C | Transition into exploitation; Harold receives the letter |
|  | 70 | I > C | Harold handles the letter without opening it, appearing very sad |
|  | 87 | I > C | Harold tears up the letter without reading it |
| 6 – Mistaken Sheriff Identity | 1 | I > C | Two men discuss how to obtain a permit |
|  | 2 | I > C | Two men discuss how to obtain a permit |
|  | 77 | I > C | One of the men persuades Harold to sign the permit |
|  | 184 | C > I | The man turns to his colleague and winks, indicating that he has obtained the signature. |

*Note*. Frames were identified from clip-specific linear mixed-effects analyses showing significant condition differences in self-paced reaction times (Installation vs. Control; p < .05, corrected). Direction indicates the condition with longer reaction times (I > C = longer in Installation; C > I = longer in Control).
